# Crocus sativus L.-derived lauric acid-functionalized gold nanoparticles induce ferroptosis in HeLa cells and reverse M2 macrophage polarization via the PGE2/EP2/cAMP-PKA signaling pathway: a network pharmacology-based study

**DOI:** 10.3389/fphar.2026.1863160

**Published:** 2026-07-07

**Authors:** Jie Weng, Songlian Liu, Yajun Tong, Kewei Tang, Leilan Yin, Site Bai, Ludi Ou, Qinghua Yin, Qiang Zhou

**Affiliations:** 1 Department of Oncology/Institute of Cancer Prevention and Intervention, Yueyang Central Hospital/ Yueyang Hospital of Xiangya School of Medicine, Central South University, Yueyang, Hunan, China; 2 Key Laboratory for Cervical Cancer Prevention and Control, Yueyang, Hunan, China

**Keywords:** cervical cancer, Crocus sativus L., ferroptosis, gold nanoparticles, lauric acid, network pharmacology, PGE2/EP2 signaling pathway, tumor-associated macrophages

## Abstract

**Introduction:**

Cervical cancer ranks as the fourth most common malignancy among women worldwide. It is closely associated with the dysregulation of numerous genes and signaling pathways. Conventional treatments for cervical cancer often lead to adverse side effects and the development of drug resistance.

**Methods:**

In this study, network pharmacology was employed to identify the active components and potential targets of Crocus sativus L. Molecular docking and surface plasmon resonance analyses were used to validate the interaction between lauric acid and a key target. Lauric acid‐modified gold nanoparticles (Au@LA) were synthesized and characterized.

**Results:**

Twenty‐three active components and 819 potential targets of Crocus sativus L. were identified, with PTGS2 being the main target. Au@LA selectively inhibited HeLa cell proliferation, induced apoptosis, downregulated SLC7A11 and GPX4, and modulated oxidative stress markers. In macrophages, Au@LA shifted M2 polarization to the pro‐inflammatory M1 phenotype and modulated the PGE2/EP2/cAMP‐PKA signaling axis.

**Conclusion:**

Our findings indicate that Au@LA has dual anti‐cervical cancer properties through inducing ferroptosis in HeLa cells and reprogramming macrophage polarization. It shows promise as a candidate for natural product‐based nanomedicine in cervical cancer therapy, potentially opening new avenues for more effective and targeted treatments in the future.

## 1 Introduction

Cervical cancer is one of the most common malignancies among women worldwide, with persistently high morbidity and mortality rates, posing a serious threat to women’s health (Han et al., 2024; Siegel et al., 2025). Current clinical treatments rely primarily on surgery, radiotherapy, and chemotherapy; however, these approaches are often accompanied by drug resistance, recurrence, and side effects (Yadav et al., 2023). Consequently, developing novel therapeutic strategies, particularly targeted therapies based on natural products, has become an important direction in cancer research (Zein et al., 2022; Hamza et al., 2023; Thanikachalam et al., 2025).

Crocus sativus L (saffron) is a valuable traditional Chinese medicine and spice, widely used in traditional medicine to treat inflammation, depression, menstrual disorders, and cardiovascular diseases (Azami et al., 2021; Abu-Izneid et al., 2022; Singh and Sharma, 2025). Modern pharmacological studies have demonstrated that Crocus sativus L. possesses various bioactivities, including anti-tumor, antioxidant, and immunomodulatory effects (Lambrianidou et al., 2020; Poursamimi et al., 2020; Ziani et al., 2025). However, the composition of Crocus sativus L. is complex, and its mechanism of action in treating diseases has not been fully elucidated. Moreover, its main active components (such as crocin, safranal, and lauric acid) suffer from poor solubility and low bioavailability, limiting their clinical translation (Ali et al., 2022; Nasrpour et al., 2022; Soltani et al., 2024).

Network pharmacology, a systems biology approach, can reveal the pharmacological mechanisms of traditional Chinese medicines from a “multi-component, multi-target, multi-pathway” holistic perspective, aligning well with the holistic view of traditional Chinese medicine (Wang et al., 2021; Wu et al., 2022; Liang et al., 2025). In recent years, network pharmacology has been successfully used to predict active components and targets of various herbs and natural products, providing a theoretical basis for experimental validation (Zhai et al., 2025). Based on this, this study first employed network pharmacology to screen the active components of Crocus sativus L., predict their targets and enriched pathways, and construct a “component-target-pathway-disease” network to identify key active components and core targets.

Lauric acid (LA), a medium-chain saturated fatty acid and a constituent of Crocus sativus L., exhibits antibacterial, anti-inflammatory, and anti-tumor activities (Mallick and Mondal, 2023; Figueiredo et al., 2025). However, it is extremely poor water solubility and low *in vivo* bioavailability severely limit its application (Al-Akayleh et al., 2020; Yeo et al., 2022; Figueiredo et al., 2025). Nano-delivery systems, particularly gold nanoparticles (Au NPs), have become effective strategies to improve the bioavailability of poorly soluble drugs due to their good biocompatibility, tunable surface chemistry, and ease of functionalization (Vashitha and Khan, 2025). Conjugating lauric acid to the surface of gold nanoparticles can improve its solubility, stability, and cellular uptake efficiency, thereby enhancing its anti-tumor effects.

Ferroptosis is an iron-dependent, lipid peroxidation-driven form of regulated cell death characterized by glutathione depletion, inactivation of glutathione peroxidase 4 (GPX4), accumulation of lipid reactive oxygen species, and iron overload (Jiang et al., 2021). Inducing ferroptosis in tumor cells has recently emerged as a promising anti-cancer strategy (Jiang et al., 2020; Zhou et al., 2024). Network pharmacology predictions suggested that Crocus sativus L. targets are significantly enriched in pathways related to “long-chain fatty acid metabolism” and “ferroptosis”, with prostaglandin-endoperoxide synthase 2 (PTGS2/COX-2) being one of the core targets. PTGS2 is the rate-limiting enzyme in the synthesis of prostaglandin E2 (PGE2). The PGE2/EP2/cAMP-PKA signaling axis not only promotes tumor cell proliferation and apoptosis resistance but also drives the polarization of tumor-associated macrophages toward an immunosuppressive M2-like polarization (Dean and Hooks, 2022). Therefore, based on the network pharmacology predictions, we hypothesized that lauric acid might exert its effects through PTGS2 to modulate ferroptosis and the PGE2-mediated immune microenvironment.

Tumor-associated macrophages (TAMs) in the tumor microenvironment are predominantly polarized to the M2-like polarization, promoting tumor growth, metastasis, and immune escape (Yang et al., 2020; Tang et al., 2025). Reversing M2 macrophage polarization toward the M1 phenotype is an important strategy for remodeling the tumor immune microenvironment (Shan et al., 2020; Zhang J. Y. et al., 2024; Zhang S. et al., 2024). However, the effects of lauric acid on macrophage polarization and its molecular mechanisms remain unclear.

Despite recent advances in understanding the anti-cancer properties of lauric acid, several critical gaps remain. First, the direct anti-cervical cancer activity of lauric acid and its molecular targets are largely unknown, as most studies have focused on its antimicrobial or anti-inflammatory effects. Second, the poor aqueous solubility of lauric acid severely limits its bioavailability, and while various nanoformulations have been explored for other fatty acids, lauric acid-specific delivery systems for cancer therapy remain underdeveloped. Third, existing gold nanoparticle-based strategies for cervical cancer primarily rely on photothermal effects or conventional chemotherapy loading, whereas the intrinsic bioactivity of surface-modified fatty acids has not been systematically exploited. Fourth, although ferroptosis induction is an emerging anticancer strategy, most reported ferroptosis inducers are synthetic small molecules with potential toxicity; natural product-derived inducers with dual functions on cancer cells and immune modulation are rare. This study aims to address these gaps by identifying lauric acid as a potential anti-cervical cancer component of Crocus sativus L., developing a lauric acid-modified gold nanoparticle (Au@LA) to improve its delivery, and evaluating its dual ferroptosis-inducing and immunomodulatory effects.

In summary, this study aimed to: (1) predict the key active components and core targets of Crocus sativus L. for disease treatment using network pharmacology; (2) prepare lauric acid-functionalized gold nanoparticles (Au@LA) and characterize their physicochemical properties; (3) verify at the cellular level whether Au@LA induces ferroptosis in HeLa cells and reverses M2 macrophage polarization via the PTGS2/PGE2/EP2/cAMP-PKA signaling axis; and (4) elucidate the dual regulatory effects of Au@LA on tumor cells and the immune microenvironment, providing a theoretical basis and a novel nanoformulation strategy for the anti-tumor application of Crocus sativus L.-derived lauric acid.

## 2 Materials and methods

### 2.1 Network pharmacology-based analysis of the anti-cervical cancer mechanism of Crocus sativus L

#### 2.1.1 Screening of active components

The bioactive components of Crocus sativus L. were retrieved from the Traditional Chinese Medicine Systems Pharmacology Database and Analysis Platform (TCMSP, https://tcmspw.com/tcmsp.php). Drug-likeness filtering was performed using Lipinski’s rule of five with oral bioavailability (OB) > 30% and quantitative estimate of drug-likeness (QED) > 0.5 as criteria. The ADMET-AI platform (https://admet.ai.greenstonebio.com/) was used to predict the bioavailability and drug-like properties of candidate components. The final list of active components is provided in Supplementary S1.

#### 2.1.2 Prediction of active component targets

Potential targets of the screened active components were predicted using the TCMSP, ChEMBL (https://www.ebi.ac.uk/chembl/), and STITCH (http://stitch.embl.de/) databases. For ChEMBL, targets with a confidence threshold >6 and an activity annotation of “active” or “both” were selected. For STITCH, targets with interaction scores >300 were selected. All targets were mapped to official gene names using the UniProt database (https://www.uniprot.org/). The complete list of predicted active component targets is provided in Supplementary S2.

#### 2.1.3 Acquisition of disease-related targets

Disease-related targets for “cervical cancer” were retrieved from the GeneCards (https://www.genecards.org/), OMIM (https://www.omim.org/), and TTD (https://idrblab.net/ttd/) databases. The GeneCards database was accessed on 30 September 2025, using the exact query term “Cervical Cancer”. Initially, 49,991 targets were obtained from GeneCards (with a minimum relevance score of 0.48, and no cut-off value was applied), plus additional targets from OMIM and TTD, giving a total of 50,003 disease-related targets. No sensitivity analysis was performed. All targets were directly downloaded without additional filtering. Duplicate targets were removed, and only human genes were top 10 bioactive compoundsretained.

#### 2.1.4 Venn diagram analysis and intersection targets

The R package VennDiagram (version 1.6.20) was used to generate Venn diagrams showing the intersections among active component targets from different databases and between active component targets and disease targets. Overlapping targets were considered potential targets for Crocus sativus L. in treating cervical cancer.

#### 2.1.5 GO functional enrichment and KEGG pathway enrichment analyses

Gene Ontology (GO) functional enrichment analysis, including biological processes (BP), cellular components (CC), and molecular functions (MF), as well as Kyoto Encyclopedia of Genes and Genomes (KEGG) pathway enrichment analysis, were performed on the intersection targets using the Python package gseapy (version 0.10.4). The background genome was set to the human reference genome. A false discovery rate (FDR)-adjusted P value <0.05 was considered statistically significant. Visualization was performed using bar plots and bubble plots generated with ggplot2 (version 3.3.5).

#### 2.1.6 Protein-protein interaction (PPI) network construction

The intersection target proteins were submitted to the STRING database (version 11, https://string-db.org/), and protein-protein interaction data with a confidence score ≥0.7 (high confidence) were selected. The PPI network was constructed and visualized using the R packages network and ggplot2. The degree of each node was calculated, and targets with high degree values were identified as core targets. When the number of intersection targets was ≥100, the top 100 targets by degree were displayed; in this study, as the number was <100, the top 10 targets were displayed based on actual conditions.

#### 2.1.7 “Component-target-pathway-disease” network construction

A multi-layer network integrating active components, targets, KEGG pathways, and the disease was constructed. The top 30 active components by degree value, the top 10 most significant KEGG pathways (by adjusted P value), and their corresponding targets were selected and visualized using the R packages network and ggrepel. Concurrently, a “component-target-GO” interaction network was constructed based on the top 10 GO terms.

#### 2.1.8 Molecular docking validation

Molecular docking between the screened core compounds and the core target protein with the highest degree value from the PPI analysis was performed using AutoDock Vina (version 1.1.2). Three-dimensional structures of target proteins were downloaded from the AlphaFold database (https://alphafold.ebi.ac.uk/), and three-dimensional SDF structures of ligands were downloaded from the PubChem database (https://pubchem.ncbi.nlm.nih.gov/). Potential protein binding sites were predicted using RDkit. Binding free energies (kcal/mol) were recorded. The best docking poses were selected, and three-dimensional and two-dimensional interaction diagrams were generated using py3Dmol, MDAnalysis, prolif, and RDkit.

#### 2.1.9 Surface plasmon resonance (SPR) assay

SPR assays were performed to test the interactions between the target protein and the active component using a Biacore 1 K+ (GE Healthcare) with a CM5 sensor chip (GE Healthcare) at 25 °C. The running buffer was PBST (10 mM Na_2_HPO_4_, 2 mM KH_2_PO_4_, pH 7.4, 137 mM NaCl, 2.7 mM KCl, 0.05% Tween 20). The target protein was pre-immobilized onto the CM5 sensor chip to a level of approximately 1900 resonance units (RU) using standard amine coupling chemistry at a concentration of 40 μg/mL. A series of concentration gradients of the active component were flowed over the chip to evaluate binding.

### 2.2 Preparation of lauric acid-modified gold nanoparticles (Au@LA)

#### 2.2.1 Preparation of gold nanoparticles (au NPs)

Gold nanoparticles were prepared using the sodium citrate reduction method. Briefly, 1 g of chloroauric acid trihydrate (HAuCl_4_·H_2_O, Macklin, China) was dissolved in 100 mL of ultrapure water and filtered to remove glass debris, yielding a 1% (w/v) HAuCl_4_ stock solution, stored at 4 °C in the dark. One milliliter of the stock solution was added to 95 mL of ultrapure water and heated to boiling in a 130 °C oil bath. Under vigorous stirring, 4 mL of 1% (w/v) sodium citrate solution was rapidly added in one portion, and heating with vigorous stirring was continued for 30 min. The reaction mixture gradually changed from pale yellow to wine red. After natural cooling to room temperature, the mixture was centrifuged at 12,000 rpm for 30 min at 4 °C to remove free sodium citrate. The precipitate was resuspended in ultrapure water and adjusted to a final volume of 100 mL, stored at 4 °C in the dark.

#### 2.2.2 Preparation of amino-modified gold nanoparticles (Au-NH_2_)

To 100 mL of the above Au NP dispersion, 1 µM cysteamine hydrochloride (Aladdin, China) was added dropwise at a molar ratio of 20:1 (Au:cysteamine), with gentle swirling during addition to prevent local concentration-induced aggregation. The mixture was incubated overnight in a shaker at 37 °C with mild agitation to allow the thiol group of cysteamine to displace citrate on the gold surface. After the reaction, the mixture was centrifuged at 12,000 rpm for 30 min at 4 °C to remove free cysteamine. The precipitate was dried as much as possible and then resuspended in 2 mL of anhydrous ethanol. If residual water was present, the sample was briefly dried in an oven for 1–2 h, avoiding prolonged drying to prevent aggregation.

#### 2.2.3 Preparation of lauric acid-modified gold nanoparticles (Au@LA)

Ten grams of lauric acid (LA, Macklin, China) was placed in a round-bottom flask and heated in a 50 °C oil bath until completely melted into a transparent liquid. Two milliliters of the above Au-NH_2_ dispersion in anhydrous ethanol was rapidly added to the liquid lauric acid, with the aid of a pre-warmed ultrasonic cleaner for dispersion (the operation must be rapid as lauric acid solidifies quickly at room temperature). The reaction was stirred at 50 °C overnight. After the reaction, 5 mL of anhydrous ethanol was added, and the mixture was centrifuged at 12,000 rpm for 30 min at 4 °C. The supernatant was discarded, and the wash was repeated three times to remove unbound lauric acid. The final precipitate was resuspended in ultrapure water to obtain the Au@LA nanoparticle dispersion. The supernatant from each centrifugation was collected, and ethanol was evaporated overnight in a 45 °C oven. The remaining liquid lauric acid was solidified at room temperature and weighed to calculate the mass of lauric acid loaded onto the gold nanoparticle surface.

#### 2.2.4 Characterization methods

UV-visible spectrophotometry (UV-Vis, Shimadzu, Japan) was used to measure the absorption spectra of Au NPs and Au@LA over a scan range of 400–800 nm. The zeta potential of the samples was measured using a Malvern nano-size analyzer. The morphology of the nanoparticles was observed using transmission electron microscopy (TEM, JEOL, Japan), and the size distribution of at least 200 particles was statistically analyzed. The hydrodynamic diameter and polydispersity index (PDI) were determined by dynamic light scattering (DLS).

### 2.3 Safety evaluation of lauric acid coated gold nanoparticles (Au@LA)

#### 2.3.1 Hemolysis assay

The hemocompatibility of lauric acid coated gold nanoparticles (Au@LA) was evaluated by a hemolysis test using fresh mouse blood. Blood was collected from healthy C57BL/6 mice into tubes containing sodium citrate (3.8%, 1:9 v/v). Au@LA was dissolved or dispersed in sterile saline at different concentrations. For the hemolysis assay, 100 µL of diluted blood (blood:saline = 1.25:1, v/v) was mixed with 1 mL of the Au@LA solution and incubated at 37 °C for 60 min. Distilled water and saline were used as positive and negative controls, respectively. After incubation, the mixtures were centrifuged at 1,000 *g* for 5 min, and the absorbance of the supernatant was measured at 540 nm. The hemolysis rate (%) was calculated as (A_sample − A_negative)/(A_positive − A_negative) × 100%.

#### 2.3.2 *In vivo* organ toxicity evaluation

All animal procedures were approved by the Ethics Committee of Jishou University. Specific pathogen-free (SPF) male C57BL/6 mice aged 6–8 weeks and weighing 18–22 g were obtained from an authorized laboratory animal center. The mice were acclimatized for 3–5 days under standard conditions (12 h light/dark cycle, 22 °C ± 2 °C, 50% ± 10% humidity) with free access to food and water. After acclimation, the mice were randomly divided into two groups (n = X per group): a control group (saline) and an Au@LA treatment group. Au@LA was administered via tail vein injection at the indicated concentration once daily for 28 consecutive days. At the end of the treatment period, all mice were sacrificed under deep anesthesia (e.g., 1% pentobarbital sodium, 50 mg/kg, i. p.). The lungs, liver, heart, kidneys, and spleen were immediately collected, washed with cold PBS, and fixed in 4% paraformaldehyde solution for at least 24 h. After fixation, tissues were dehydrated through graded ethanol, embedded in paraffin, sectioned at 5 µm thickness, and stained with hematoxylin and eosin (H&E). Histopathological examination was performed under a light microscope (e.g., Olympus BX53) by a pathologist blinded to the group allocation. Any signs of inflammation, necrosis, congestion, or other morphological abnormalities were recorded and photographed. This systematic evaluation was used to assess the *in vivo* biosafety of Au@LA.

### 2.4 *In Vitro* cell experiments

#### 2.4.1 Cell culture and drug treatment

The human cervical cancer HeLa cell line was purchased from ATCC (CCL-2). Cells were cultured in DMEM/F12 medium containing 10% fetal bovine serum (FBS, Hangzhou Tianhang) and 1% penicillin-streptomycin at 37 °C in a 5% CO_2_ incubator. When cells reached 80%–90% confluence, they were passaged using 0.25% trypsin-EDTA. Before drug treatment, HeLa cells were starved for 12 h in serum-free basal medium containing 1% bovine serum albumin (BSA). Subsequently, the medium was replaced with complete medium containing different concentrations of monomers or metal materials (e.g., Au@LA), and treatment continued for 48 h. Control groups received an equal volume of control solvent.

The human normal cervical immortalized epithelial cell line Ect1/E6E7 was purchased from ATCC (CRL-2614™). Cells were cultured in keratinocyte serum-free medium (K-SFM, Gibco™) supplemented with 0.1 ng/mL human recombinant epidermal growth factor (EGF), 0.05 mg/mL bovine pituitary extract (BPE), and 0.4 mM calcium chloride (CaCl_2_) at 37 °C in a 5% CO_2_ incubator. When cells reached 80%–90% confluence, they were passaged using 0.05% trypsin-EDTA. Before drug treatment, Ect1/E6E7 cells were starved for 12 h in basal K-SFM medium without EGF and BPE. Subsequently, the medium was replaced with complete medium containing different concentrations of monomers or metal materials (e.g., Au@LA), and treatment continued for 48 h. Control groups received an equal volume of control solvent.

THP-1 cells (YouSi Bio, YLH147) were cultured in RPMI-1640 medium containing 10% fetal bovine serum and 1% penicillin-streptomycin. To induce differentiation into macrophages, THP-1 cells were seeded in 6-well plates at a density of 5 × 10^5^ cells/mL and treated with 100 ng/mL phorbol 12-myristate 13-acetate (PMA, MCE, HY-18739) for 48 h. Differentiated cells became adherent and were washed twice with PBS (GenoBio, GNM20012) to remove non-adherent cells, yielding THP-1-derived macrophages. Subsequently, cells were treated with different drugs (e.g., monomers or metal materials) for 48 h according to the experimental design. After treatment, cells were collected for subsequent assays.

#### 2.4.2 CCK-8 cell proliferation assay

Cells in the logarithmic growth phase were seeded in 96-well plates at 1 × 10^4^ cells/well (100 µL per well) and cultured until adherence. Following starvation and drug treatment as described above, 100 µL of CCK-8 working solution (Beyotime, C0038) was added to each well and incubated at 37 °C for 1–4 h. Absorbance at 450 nm (OD_450_) was measured using a microplate reader. Blank wells (medium only, no cells) and control wells (solvent-treated) were set. The cell proliferation rate (%) was calculated as (experimental OD − blank OD)/(control OD − blank OD) × 100%. Three replicate wells were used for each concentration, and the experiment was independently repeated three times.

#### 2.4.3 Colony formation assay

Cells in the logarithmic growth phase were treated with drugs for 48 h, then trypsinized and reseeded into 6-well plates at 500 cells per well, and cultured for 14 days. The medium containing corresponding drugs was replaced every 3 days. When visible colonies appeared, the medium was discarded, cells were washed twice with PBS, fixed with 4% paraformaldehyde for 20 min, and stained with 0.1% crystal violet for 15 min. After washing with water and air drying, colonies containing ≥50 cells were counted under a microscope. The colony formation rate (%) = (number of colonies in experimental group/number of colonies in control group) × 100%.

#### 2.4.4 Flow cytometry for apoptosis detection

Apoptosis was detected using the Annexin V-FITC/PI double staining method. After 48 h of drug treatment, cells (including suspended cells in the supernatant) were collected, washed twice with PBS, and centrifuged at 300 *g* for 5 min. Cells were resuspended in 100 µL of 1× Binding Buffer, and 5 µL of Annexin V-FITC and 5 µL of PI (Beyotime, C1062 S) were added, followed by incubation for 15 min at room temperature in the dark. Then, 400 µL of Binding Buffer was added, and cells were analyzed within 1 h using a flow cytometer (Beckman CytoFLEX). FlowJo software was used to analyze early apoptotic (Annexin V^+^/PI^−^), late apoptotic (Annexin V^+^/PI^+^), and total apoptotic rates.

#### 2.4.5 Flow cytometry for macrophage polarization

THP-1-derived macrophages after drug treatment were collected, washed twice with PBS (GenoBio, GNM20012), centrifuged at 300 *g* for 5 min, and resuspended in 100 µL of fixation buffer, followed by incubation for 20 min at 4 °C in the dark. After washing once with PBS, cells were resuspended in 100 µL of permeabilization buffer for staining of M1 and M2 markers. For flow cytometry staining, cells were divided into two separate tubes: one for M1 marker detection and the other for M2 marker detection. For M1 detection, FITC labeled mouse anti human CD68 antibody (Invitrogen, 11–0,689–41, 5 µL/test) and rabbit anti human iNOS primary antibody (Abcam, ab283655, 1:100 dilution) were added simultaneously and incubated for 1 h at 4 °C in the dark. After two PBS washes, Alexa Fluor® 647 conjugated goat anti rabbit IgG secondary antibody (Abcam, ab150083) was added and incubated for 30 min at 4 °C in the dark. After two washes, corresponding isotype controls (FITC labeled mouse IgG2b, Invitrogen, 11–4,732–81; and rabbit IgG, Abcam, ab172730) were also used. For M2 detection, FITC-labeled mouse anti-human CD68 antibody (Invitrogen, 11–0,689–41, 5 µL/test) and PE-labeled mouse anti-human Arg-1 antibody (Invitrogen, 12–3,697–82, 2.5 µL/test) were added simultaneously and incubated for 1 h at 4 °C in the dark. Corresponding isotype controls, FITC-labeled mouse IgG2b (Invitrogen, 11–4,732–81) and PE-labeled rat IgG2a (Invitrogen, 12–4,321–80), were also used. After staining, cells were washed twice with PBS, centrifuged at 300 *g* for 5 min, resuspended in 100 µL PBS, and analyzed using a flow cytometer (Beckman CytoFLEX). THP-1-derived macrophages were gated using forward scatter (FSC) and side scatter (SSC), and at least 10,000 cells were collected per sample. Isotype controls and unstained cells were used to set positive/negative gates. Results are expressed as the percentage of CD68^+^/iNOS^+^ cells for M1 macrophages and the percentage of CD68^+^/Arg-1^+^ cells for M2-like polarization.

#### 2.4.6 Enzyme-linked immunosorbent assay (ELISA)

Cell culture supernatants were collected and centrifuged at 300 *g* for 5 min to remove cell debris. The supernatants were used for detection. ELISAs were performed according to the manufacturers’ instructions to detect human IL-10 (YouSi Bio, YE18956), IFN-γ (YouSi Bio, YE18850), PGE2 (YouSi Bio, YE28811), 4-hydroxynonenal (4-HNE, YouSi Bio, YE17928), and cAMP (YouSi Bio, YE25930). Briefly, standards or samples (100 µL per well) were added to pre-coated plates and incubated at 37 °C for 90 min. After washing, biotinylated antibody working solution was added and incubated at 37 °C for 60 min. After another wash, HRP enzyme conjugate working solution was added and incubated at 37 °C for 30 min. Following thorough washing, TMB substrate solution was added and incubated at 37 °C in the dark for 15 min. Stop solution was added, and the absorbance at 450 nm was immediately read using a microplate reader. Standard curves were plotted, and sample concentrations were calculated. Each sample was assayed in duplicate, and results are expressed as pg/mL or ng/mL.

#### 2.4.7 Biochemical indicator detection

Adherent cells after drug treatment were collected, washed twice with pre-chilled PBS, and lysed in RIPA lysis buffer (ASPEN) containing protease inhibitors on ice for 30 min. The lysate was centrifuged at 12,000 × g for 5 min at 4 °C, and the supernatant was used for biochemical assays and protein quantification. Malondialdehyde (MDA) content was detected using the thiobarbituric acid (TBA) method according to the kit instructions (Nanjing Jiancheng, A003-1), with absorbance measured at 532 nm. Reduced glutathione (GSH, Nanjing Jiancheng, A006-2) and oxidized glutathione (GSSG, Nanjing Jiancheng, A061-1) were detected using microplate methods, with absorbance measured at 405 nm, and GSH and GSSG concentrations were calculated. Ferrous ion (Fe^2+^, Elabscience, E-BC-K881-M) and total iron (Elabscience, E-BC-K880-M) contents were detected using colorimetric methods, with absorbance measured at 593 nm after adding corresponding probes or reducing agents; Fe^3+^ content was calculated as total iron minus Fe^2+^. All biochemical indicators were normalized to protein concentration determined using a BCA protein assay kit (ASPEN) on the same batch of samples, and results are expressed as content per milligram of protein (e.g., μmol/mg protein).

#### 2.4.8 Reactive oxygen species (ROS) assay

Intracellular reactive oxygen species (ROS) levels were assessed using the cell-permeable fluorescent probe 2′,7′-dichlorodihydrofluorescein diacetate (DCFH-DA, Sigma-Aldrich, D6883). Cells were seeded in 24-well plates containing sterile coverslips at a density of 1 × 10^5^ cells/well and cultured overnight. After treatment with different concentrations of the active component or vehicle control for the indicated time, the culture medium was removed, and cells were gently washed once with pre-warmed PBS. Fresh serum-free medium containing 10 µM DCFH-DA was added to each well, and the cells were incubated at 37 °C in the dark for 30 min. Following incubation, cells were washed twice with PBS to remove excess probe. Cells were then fixed with 4% paraformaldehyde for 15 min at room temperature, washed twice with PBS, and counterstained with DAPI (1 μg/mL, Sigma-Aldrich, D9542) for 10 min at room temperature in the dark. Coverslips were mounted onto glass slides with antifade mounting medium (Solarbio, S2100). Fluorescence images were captured using a fluorescence microscope (Olympus IX73) equipped with a 488 nm excitation filter for DCF (green fluorescence) and a 405 nm excitation filter for DAPI (blue fluorescence). At least five random fields per well were photographed under the same exposure settings. The mean fluorescence intensity of DCF was quantified using ImageJ software (NIH), and results were expressed as fold change relative to the untreated control group.

#### 2.4.9 Western blot analysis

Adherent cells (HeLa or THP-1-derived macrophages) after drug treatment were washed twice with pre-chilled PBS and lysed in RIPA lysis buffer (ASPEN) containing protease and phosphatase inhibitors on ice for 30 min. The lysate was centrifuged at 12,000 × g for 5 min at 4 °C, and the supernatant was collected. Protein concentration was determined using a BCA kit. Forty micrograms of total protein were mixed with 5× protein loading buffer and boiled at 95 °C for 5 min. Proteins were separated by 8%–12% SDS-PAGE (80 V in stacking gel, 120 V in separating gel) and transferred to PVDF membranes (Millipore, 0.45 μm) using a wet transfer method (300 mA constant current). Transfer time was adjusted according to the molecular weight of the target protein (typically 1 min per kDa, e.g., 55 min for 55 kDa). After transfer, membranes were blocked with 5% non-fat milk (in TBST) for 1 h at room temperature. Primary antibodies were as follows: rabbit anti-human SLC7A11 (Abcam, ab175186, 1:1,000), rabbit anti-human GPX4 (Abcam, ab125066, 1:1,000), rabbit anti-human TFR1 (Abcam, ab214039, 1:1,000), rabbit anti-human EP2 receptor (PTGER2, CST, 10135-1-AP, 1:500), rabbit anti-human CREB (CST, 9,197, 1:1,000), rabbit anti-human p-CREB (Ser133, CST, 9,198, 1:1,000), rabbit anti-human PKA Catalytic Subunit (CST, 4,782, 1:1,000), rabbit anti-human p-PKA (Thr197, CST, 5,661, 1:500), and mouse anti-human GAPDH (Proteintech, 60004-1-Ig, 1:5,000). Primary antibodies were incubated overnight at 4 °C. The next day, membranes were washed three times with TBST for 5 min each, then incubated with HRP-conjugated goat anti-rabbit or anti-mouse secondary antibodies (ASPEN, 1:5,000) for 30 min at room temperature, followed by four TBST washes. ECL chemiluminescent substrate (ASPEN) was applied, and membranes were exposed to X-ray film in the dark. Scanned film bands were analyzed using ImageJ software, and band intensities were normalized to GAPDH as an internal control.

### 2.5 Statistical analysis

All cell culture experiments were independently repeated at least three times using separate cell passages or independent nanoparticle preparations; thus, n = 3 represents the number of independent biological replicates for all quantitative cell-based assays (e.g., CCK-8, flow cytometry, Western blot, ELISA, biochemical assays). For each biological replicate, technical duplicates or triplicates were averaged before statistical analysis. For animal experiments, only histopathological examination was performed without quantitative endpoint measurements; therefore, no statistical comparison was applied to the animal data. All quantitative data are presented as mean ± standard deviation (mean ± SD) from at least three independent biological replicates. GraphPad Prism 9.0 software was used for statistical analysis. Comparisons between two groups were performed using two-tailed unpaired Student’s t-test. Comparisons among three or more groups were performed using one-way analysis of variance (ANOVA) followed by Tukey’s *post hoc* test for multiple comparisons. All P values are reported as inequality thresholds. Throughout the manuscript, asterisks are used in figures and legends to denote significance levels (*P < 0.05, **P < 0.01, ***P < 0.001). A significance threshold of P < 0.05 was applied for all analyses.

## 3 Results

### 3.1 Network pharmacology predicts key targets and pathways of Crocus sativus L. In treating cervical cancer

**FIGURE 1:**
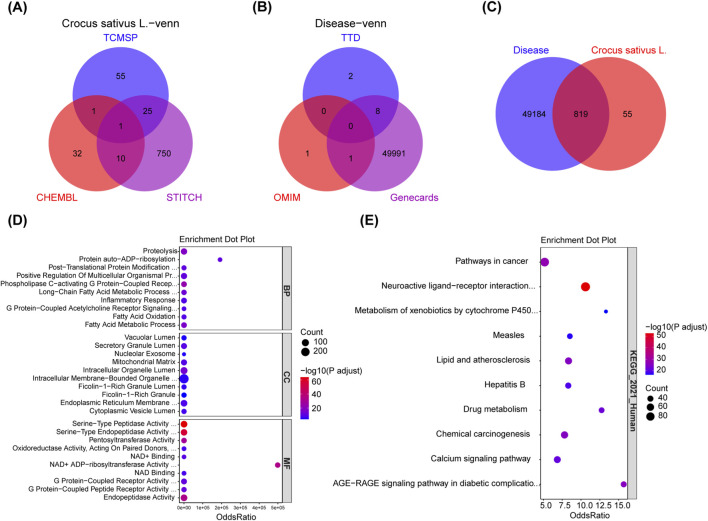
Network pharmacology predicts the key targets and pathways of *Crocus sativus* L. in treating cervical cancer. **(A)** Venn diagram showing the overlap of predicted drug targets from the TCMSP, ChEMBL, and STITCH databases. **(B)** Venn diagram showing the overlap of cervical cancer-related targets collected from the GeneCards, OMIM, and TTD databases. **(C)** Venn diagram illustrating the intersection of drug targets and disease targets, identifying 819 common potential targets. **(D)** Gene Ontology (GO) enrichment analysis of the 819 common targets. The top 10 significantly enriched terms in Biological Process (BP), Cellular Component (CC), and Molecular Function (MF) are displayed, including key enriched biological functions such as proteolysis (GO:0006508) and phospholipase C-activating G protein-coupled receptor signaling pathway (GO:0007200). Key molecular functions include serine-type peptidase activity (GO:0008236) and NAD + ADP-ribosyltransferase activity (GO:0003950). **(E)** Kyoto Encyclopedia of Genes and Genomes (KEGG) pathway enrichment analysis of the common targets. The top 20 significantly enriched pathways are shown, including Pathways in cancer (hsa05200) and Neuroactive ligand-receptor interaction (hsa04080).

To investigate the molecular mechanism of Crocus sativus L. in treating cervical cancer, network pharmacology was first employed to screen its active components and targets. Through drug-likeness analysis (OB > 30%, QED >0.5, meeting Lipinski’s rule of five) using the TCMSP database combined with the ADMET-AI platform, a total of 23 potential active components were identified (Table 1). Subsequently, the targets of these components were predicted in the TCMSP, ChEMBL (threshold >6), and STITCH (score >300) databases, yielding 874 drug targets. A Venn diagram of targets from different databases (Figure 1A) showed: 55 unique targets for TCMSP, 32 for CHEMBL, 75 for STITCH, and one common target among all three. Concurrently, disease-related targets were collected from the Genecards, OMIM, and TTD databases, resulting in 50,003 disease targets. A Venn diagram of the disease databases (Figure 1B) showed: two unique targets for TTD, one for OMIM, and 49,991 for Genecards. The intersection of the 874 drug targets with the 50,003 disease targets yielded 819 common targets (Figure 1C), which are the candidate targets through which Crocus sativus L. may potentially intervene.

**TABLE 1:** Potential active compounds in *Crocus sativus L.*

Name	Smiles	Molecular_weight	QED	Bioavailability_Ma
Safrole	C=CCC1 = CC2 = C(C=C1)OCO2	162.188	0.62	0.74
Heliettin	CC(C) (C=C)C1 = CC2 = CC3 = C(C=C2OC1 = O)OC(C3)C(C) (C)O	314.381	0.70	0.78
Linalool	CC(=CCCC(C) (C=C)O)C	154.253	0.62	0.80
T-muurolol	CC1 = CC2C(CCC(C2CC1) (C)O)C(C)C	222.372	0.67	0.95
Isodecanoic acid	CC(C)CCCCCCC(=O)O	172.268	0.60	0.85
(7aR)-4,4,7a-trimethyl-6,7-dihydro-5h-benzofuran-2-one	CC1(CCCC2(C1 = CC(=O)O2)C)C	180.247	0.54	0.83
Picrocrocin deglycosylation	CC1 = C(C(CC(C1)O) (C)C)C=O	168.236	0.60	0.84
Safranal	CC1 = C(C(CC = C1) (C)C)C=O	150.221	0.52	0.81
Oxophorone	CC1 = CC(=O)CC(C1 = O) (C)C	152.193	0.53	0.87
Globulol	CC1CCC2C1C3C(C3(C)C)CCC2(C)O	222.372	0.67	0.96
Octanoic acid	CCCCCCCC(=O)O	144.214	0.58	0.76
Lauric acid	CCCCCCCCCCCC(=O)O	200.322	0.54	0.72
Abiol	COC(=O)C1 = CC = C(C=C1)O	152.149	0.61	0.44
Myristicin	COC1 = CC(=CC2 = C1OCO2)CC = C	192.214	0.69	0.83
Isorhamnetin	COC1 = C(C=CC(=C1)C2 = C(C(=O)C3 = C(C=C(C=C3O2)O)O)O)O	316.265	0.57	0.43
NCA	C1 = CC(=CN = C1)C (=O)N	122.127	0.58	0.95
Adenine nucleoside	C1 = NC2 = NC = NC(=C2N1)N	135.13	0.53	0.82
3,4-Dihydroxybenzoic acid	C1 = CC(=C(C=C1C(=O)O)O)O	154.121	0.52	0.52
Benzoic acid	C1 = CC = C(C=C1)C (=O)O	122.123	0.61	0.94
Tyrosol	C1 = CC(=CC = C1CCO)O	138.166	0.64	0.40
Pyridin-3-yl-methanol	C1 = CC(=CN = C1)CO	109.128	0.57	0.83
Kaempferol	C1 = CC(=CC = C1C2 = C(C(=O)C3 = C(C=C(C=C3O2)O)O)O)O	286.239	0.55	0.36
Kaempferide	COC1 = CC = C(C=C1)C2 = C(C(=O)C3 = C(C=C(C=C3O2)O)O)O	300.266	0.67	0.49

GO functional enrichment analysis was performed on these 819 common targets (Figure 1D). In the biological process (BP) category, significantly enriched terms included proteolysis, protein auto-ADP-ribosylation, post-translational protein modification, positive regulation of multicellular organismal process, phospholipase C-activating G protein-coupled receptor signaling, long-chain fatty acid metabolic process, inflammatory response, G protein-coupled acetylcholine receptor signaling, fatty acid oxidation, and fatty acid metabolic process. In the cellular component (CC) category, terms were mainly enriched in vacuolar lumen, secretory granule lumen, nuclear exosome, mitochondrial matrix, intracellular organelle lumen, intracellular membrane-bounded organelle lumen, ficolin-1-rich granule lumen, ficolin-1-rich granule, endoplasmic reticulum membrane, and cytoplasmic vesicle lumen. In the molecular function (MF) category, significantly enriched terms included serine-type peptidase activity, serine-type endopeptidase activity, pentosyltransferase activity, oxidoreductase activity acting on paired donors, NAD + binding, NAD + ADP-ribosyltransferase activity, NAD binding, G protein-coupled receptor activity, G protein-coupled peptide receptor activity, and endopeptidase activity. KEGG pathway enrichment analysis (Figure 1E) showed that the common targets were significantly enriched in pathways such as pathways in cancer, neuroactive ligand-receptor interaction, metabolism of xenobiotics by cytochrome P450, measles, lipid and atherosclerosis, drug metabolism, chemical carcinogenesis, calcium signaling pathway, and the AGE-RAGE signaling pathway in diabetic complications.

**FIGURE 2:**
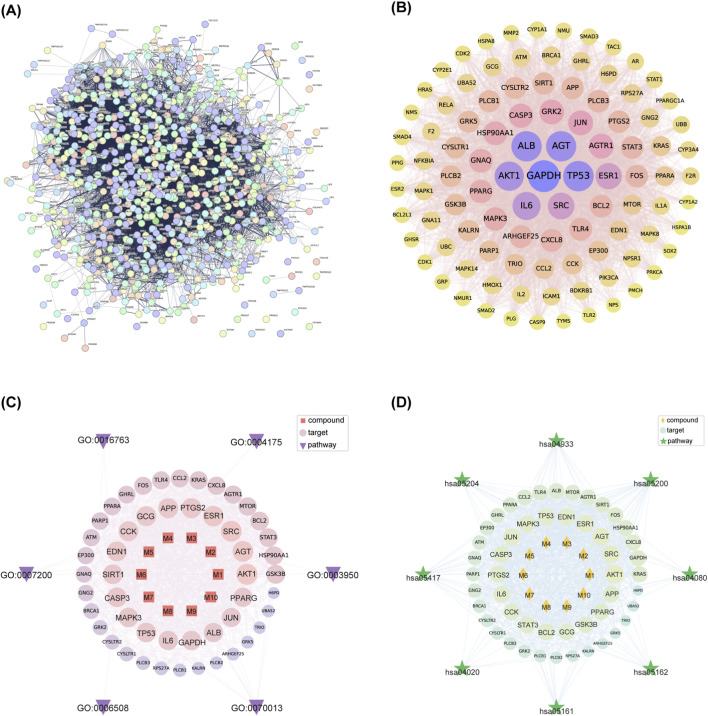
Identification of core targets and construction of the compound-target-pathway network. **(A)** Protein-protein interaction (PPI) network of the 819 common targets constructed using the STRING database (*Homo sapiens*). **(B)** The interaction network of the top 50 core targets ranked by degree value. The top 10 core targets (PTGS2, STAT3, MAPK1, MAPK3, AKT1, JUN, TNF, IL6, EGFR, VEGFA) are highlighted. **(C)** Bioactive component-target-GO network. The interaction network depicts the multi-level regulatory relationships. The nodes are arranged in three concentric layers: the innermost layer represents the top 10 bioactive compounds (e.g., M1: Lauric acid, M2: Kaempferol), the middle layer represents the 50 core target proteins, and the outermost layer represents the enriched GO terms. **(D)** Bioactive component-target-KEGG network, illustrating the connections between components, targets, and key signaling pathways.

**FIGURE 3:**
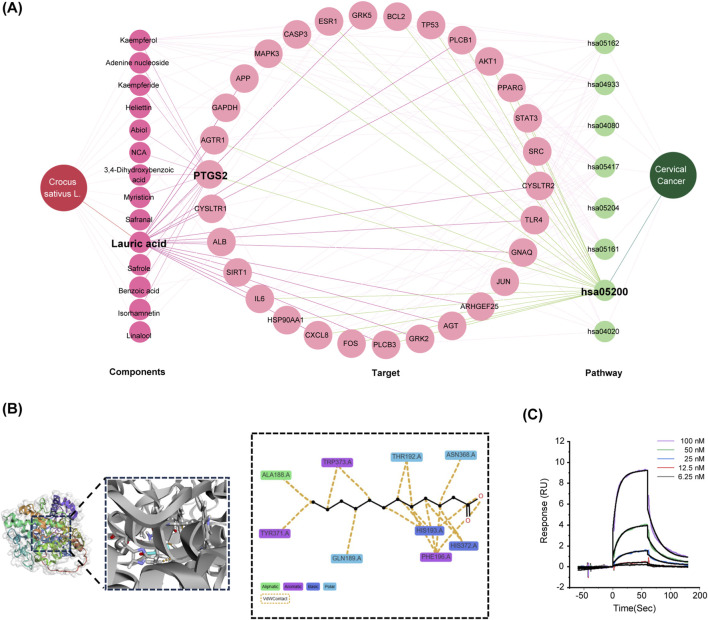
Construction of the “Component-Target-Pathway” network and molecular docking validation. **(A)** The integrative network of bioactive components, core targets, and significant KEGG pathways. The edge between Lauric acid (LA) and PTGS2 is highlighted. **(B)** Molecular docking model of lauric acid (yellow sticks) bound to the active site of PTGS2. The predicted binding energy is −4.774 kcal/mol. The color coding of the interacting residues indicates their properties. **(C)** Surface plasmon resonance (SPR) sensorgram showing the binding kinetics between lauric acid and the target protein (Response vs. Time).

A protein-protein interaction (PPI) network of the 819 target proteins was constructed based on the STRING database (Figure 2A). Topological analysis revealed significant differences in the degree values of each node. The top 50 core targets by degree value were selected, and their interaction network is shown in Figure 2B. The top 10 core targets included PTGS2, STAT3, MAPK1, MAPK3, AKT1, JUN, TNF, IL6, EGFR, and VEGFA. Using these 50 core targets as bridges, the top 10 active components (coded M1–M10; see Table 2 for code-to-name correspondence) most closely associated with them, as well as the top 10 most significant KEGG/GO pathways (by adjusted P-value), were mapped. Based on this, a three-layer network interconnecting “active components–targets–pathways” was constructed (Figures 2C,D). To visually demonstrate the overall network of Crocus sativus L.‘s multi-component, multi-target, multi-pathway synergistic regulation of the disease, a “bioactive component–target–pathway–disease” network was further constructed (Figure 3A). Network analysis revealed that components such as lauric acid, kaempferol, and isorhamnetin were located at the core hub of the network, connecting multiple high-degree targets (e.g., PTGS2, STAT3, MAPK1) and key pathways (e.g., pathways in cancer, calcium signaling pathway, AGE-RAGE pathway). Notably, a direct edge connected lauric acid and PTGS2, and PTGS2 had the highest degree value in the network, suggesting that PTGS2 may be an important candidate target based on network topology of Crocus sativus L. active components. Based on the topological features of the network, lauric acid was selected as a representative active component, and PTGS2 as the key target, for molecular docking validation. Molecular docking results (Figure 3B) showed a binding energy of −4.774 kcal/mol between lauric acid and PTGS2, with hydrophobic and van der Waals interactions forming with active site residues (THR192, ASN368, TRP373, ALA188, TYR371, GLN189), indicating a direct physical interaction between lauric acid and PTGS2 *in vitro*. To experimentally validate this interaction, Surface Plasmon Resonance (SPR) was performed (Figure 3C). Kinetic analysis demonstrated that lauric acid bound to the PTGS2 protein in a dose-dependent manner, with a calculated dissociation constant (*K*_*D*_) of approximately 6.05 × 10^−8^ M, confirming a stable and direct physical interaction.​Network pharmacology predictions also suggested that Crocus sativus L. targets are significantly enriched in pathways related to “long-chain fatty acid metabolic process,” “fatty acid oxidation,” and “ferroptosis” (Figures 1D,E). PTGS2 (cyclooxygenase-2) is a key rate-limiting enzyme in the synthesis of prostaglandin E2 (PGE2). The PGE2/EP2/cAMP-PKA signaling axis plays an important role in tumor cell proliferation, apoptosis resistance, and macrophage M2 polarization. Therefore, we hypothesized that lauric acid might exert its cellular effects through PTGS2 to regulate ferroptosis-related proteins (SLC7A11, GPX4, TFR1) and PGE2-mediated signaling pathways, thereby influencing tumor cell fate and the immune microenvironment.

**TABLE 2:** Correspondence between codes and names of active ingredients in *Crocus sativus L.*

Compounds_id	Compounds_Name	Gen_count
M1	Lauric acid	24
M2	Kaempferol	13
M3	NCA	7
M4	Adenine nucleoside	6
M5	Benzoic acid	5
M6	Isorhamnetin	4
M7	Kaempferide	3
M8	Myristicin	3
M9	Octanoic acid	3
M10	Abiol	2

### 3.2 Successful preparation and physicochemical characterization of Au@LA nanoparticles

**FIGURE 4:**
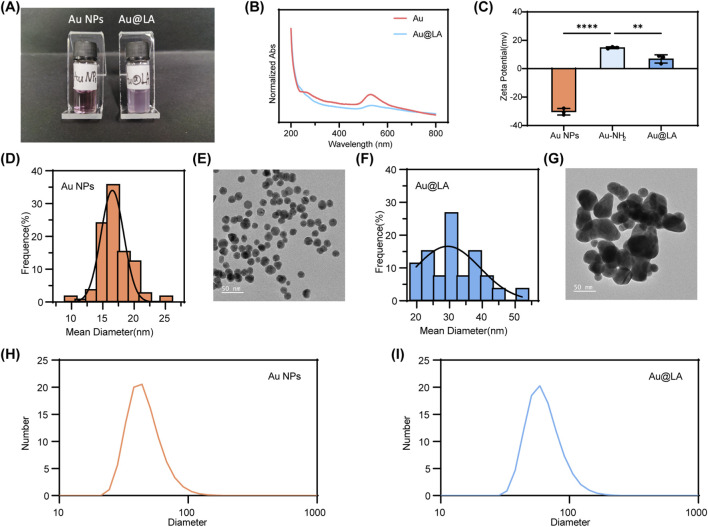
Physicochemical characterization of Au NPs and Au@LA. **(A)** Representative photographs of Au NP and Au@LA dispersions, showing the characteristic color change from wine-red to purple. **(B)** UV-Vis absorption spectra of Au NPs and Au@LA, displaying the surface plasmon resonance (SPR) peak shift. **(C)** Zeta potential measurements of Au NPs, Au-NH_2_, and Au@LA, confirming the successful surface modification. **(D,F)** Size distribution histograms of Au NPs **(D)** and Au@LA **(F)** determined by TEM analysis. **(E,G)** Representative TEM images of Au NPs **(E)** and Au@LA **(G)**. Scale bars: 50 nm. **(H,I)** Hydrodynamic size distributions of Au NPs **(H)** and Au@LA **(I)** measured by dynamic light scattering (DLS), indicating the overall size distribution in an aqueous environment.

To improve the solubility and bioavailability of lauric acid, we designed and prepared lauric acid-functionalized gold nanoparticles (Au@LA). The preparation process is illustrated in Figure 4A: gold nanoparticles (Au NPs) were first prepared by the sodium citrate reduction method, then modified with cysteamine hydrochloride to introduce amino groups (Au-NH_2_), and finally lauric acid was coupled to the gold surface, potentially via an amidation reaction. UV-visible spectroscopy (Figure 4B) showed a typical surface plasmon resonance (SPR) absorption peak for Au NPs at 529 nm, while the SPR peak for Au@LA was slightly red-shifted to 531 nm, indicating successful surface modification. Zeta potential analysis (Figure 4C) showed that Au NPs had a surface potential of −30.3 mV, attributed to residual citrate ions on the surface. After cysteamine hydrochloride modification, the potential reversed to +14.8 mV (Au-NH_2_), indicating that amino groups replaced the citrate. After further coupling with lauric acid, the potential decreased to +8.89 mV (Au@LA), indicating successful conjugation of lauric acid to the gold surface.

Transmission electron microscopy (TEM) observation (Figures 4D–G) revealed that Au NPs were uniformly spherical with an average particle size of 16.86 ± 2.53 nm and good dispersion. Au@LA particles showed some degree of aggregation, with an increased average particle size of 31.17 ± 8.44 nm (Figures 4F,G), consistent with the reduced zeta potential indicating decreased stability. Dynamic light scattering (DLS) measured the hydrodynamic diameter of Au NPs as 26.66 ± 7.14 nm and that of Au@LA as 65.58 ± 7.64 nm (Figures 4H,I), with Polydispersity index (PDI) of 0.23 and 0.28, respectively, indicating good dispersion for both nanoparticles. By gravimetric analysis, the loading concentration of lauric acid in the prepared Au@LA was calculated to be 12.5 mg/mL (dispersed in 5 mL ultrapure water, total loading amount 62.5 mg).

### 3.3 *In Vitro* hemocompatibility and preliminary in vivo safety assessment of Au@LA

**FIGURE 5:**
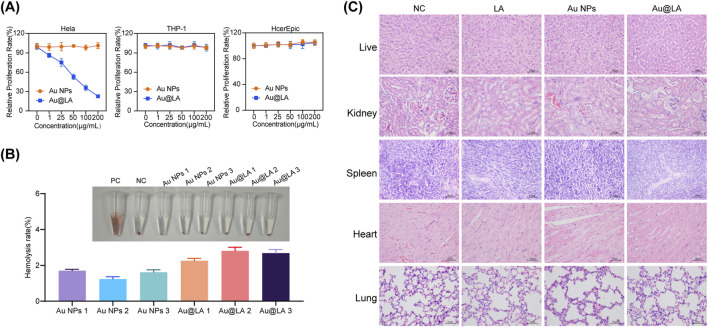
*In vitro* and *in vivo* biocompatibility and safety evaluation of Au@LA. **(A)** Relative proliferation rates of HeLa, THP-1, and HoerEpic cells treated with various concentrations of Au NPs or Au@LA for 48 h. Au@LA exhibited dose-dependent cytotoxicity against HeLa cells, while no significant cytotoxicity was observed in normal cells (THP-1 and HoerEpic) at the tested concentrations. **(B)** Hemolysis assay of Au NPs and Au@LA. The upper panel displays representative photographs of the centrifuge tubes, and the lower panel presents the quantitative analysis of hemolysis rates. Both formulations showed a hemolysis rate of less than 3%, indicating excellent blood compatibility. **(C)** Representative H&E staining images of major organs (liver, kidney, spleen, heart, and lung) collected from mice after treatment with PBS (NC), LA, Au NPs, or Au@LA. No obvious histopathological abnormalities, inflammatory infiltration, or toxic lesions were observed in any of the treated groups compared with the NC group.

Before evaluating therapeutic efficacy, we rigorously examined the biosafety of Au@LA. *In vitro* cytotoxicity screening (Figure 5A) showed no significant toxicity to normal cells at gradient concentrations of 1, 25, 100, and 200 μg/mL. *In vitro* hemocompatibility assays (Figure 5B) demonstrated that even at the highest tested concentrations (50 μg/mL, 250 μg/mL, 1,000 μg/mL), Au@LA did not cause significant erythrocyte rupture, with hemolysis rates far below the 5% clinical safety threshold, confirming its excellent hemocompatibility. *In vivo* organ toxicity assessment (Figure 5C) revealed no apparent histopathological damage or abnormal gold element accumulation in H&E-stained sections of major organs (heart, spleen, lung, kidney and live) following a single intravenous injection (10 mg/kg). These data collectively support the excellent biocompatibility of the Au@LA nanocarrier.

### 3.4 Au@LA inhibits HeLa cell proliferation and induces ferroptosis

#### 3.4.1 Effects of Au@LA on HeLa cell proliferation, apoptosis, and colony formation

**FIGURE 6:**
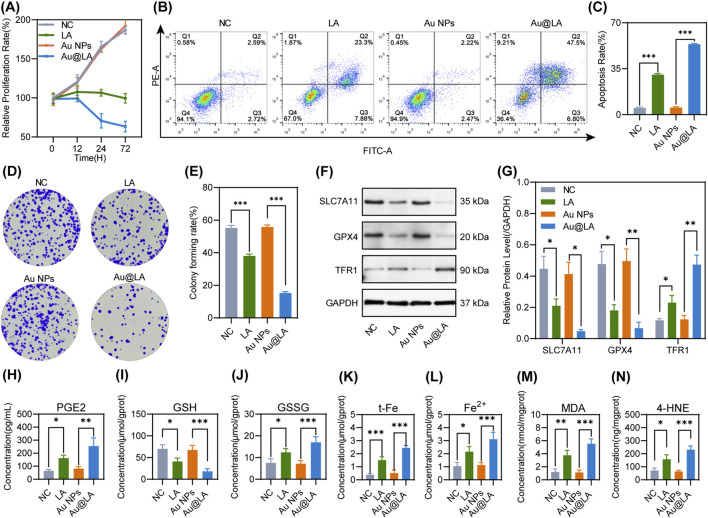
Au@LA inhibits proliferation, induces apoptosis, and triggers ferroptosis in HeLa cells. **(A)** Time-dependent proliferation rate of HeLa cells treated with LA, Au NPs, or Au@LA, as determined by the CCK-8 assay. **(B,C)** Detection of apoptosis by Annexin V-FITC/PI double staining. Representative flow cytometry dot plots **(B)** and quantitative analysis of the total apoptosis rate **(C)** are shown. **(D,E)** Colony formation assay. Representative images of HeLa cell colonies **(D)** and quantitative analysis of the colony formation rate **(E)** following the indicated treatments. **(F,G)** Western blot analysis of ferroptosis-related proteins. Representative immunoblots **(F)** and quantitative analysis **(G)** of SLC7A11, GPX4, and TFR1 protein levels, with GAPDH used as the internal loading control. Au@LA treatment resulted in a significant decrease in SLC7A11 and GPX4 levels, and a marked increase in TFR1 levels. **(H–N)** Biochemical analysis of ferroptosis markers. Measurement of intracellular levels of PGE2 **(H)**, GSH **(I)**, GSSG **(J)**, total iron (t-Fe, **(K)**, ferrous iron (Fe^2+^, **(L)**), MDA **(M)**, and 4-HNE **(N)**. Data are presented as mean ± SD (n = 3). Statistical significance is indicated by asterisks (*p < 0.05, **p < 0.01, ***p < 0.001).

The CCK-8 assay was used to evaluate the effects of different concentrations (0, 1, 25, 50, 100, 200 μM, based on lauric acid content) of Au@LA, free lauric acid (LA), and Au NPs on the proliferation of human cervical cancer HeLa cells, human normal cervical immortalized epithelial Ect1/E6E7 cells, and human monocytic THP-1 cells (Figures 6A–C). Importantly, as shown in Figure 6A, Au@LA significantly inhibited HeLa cell proliferation in a concentration-dependent manner, with a half-maximal inhibitory concentration (IC_50_) of approximately 52.29 µM (48 h). In contrast, free LA and Au NPs at the same concentration ranges showed almost no inhibitory effect on HeLa cells. Notably, Au@LA had no significant inhibitory effect on normal human cervical immortalized epithelial Ect1/E6E7 cells or human monocytic THP-1 cells (Figures 4A–C), indicating excellent selective cytotoxicity against cancer cells and good biocompatibility with normal cells, which is crucial for reducing systemic toxicity. Based on the IC_50_ results, 50 μM was selected as the uniform working concentration for LA, Au NPs, and Au@LA in all subsequent cell function experiments (equal molar amount of lauric acid), and this dose was applied in the following HeLa cell tests. In contrast, free LA and Au NPs at the same concentration (50 μM) showed almost no inhibitory effect on HeLa cells. Notably, Au@LA did not significantly inhibit normal human cervical immortalized epithelial Ect1/E6E7 cells or human monocytic THP-1 cells (Figure 6A), indicating excellent selective cytotoxicity towards cancer cells and good biocompatibility with normal cells, which is crucial for reducing systemic toxicity. The time-course proliferation curve (Figure 6A) showed that compared with the untreated NC group, the proliferation rates of HeLa cells 24 h after treatment with free LA and Au NPs were (107.72 ± 7.92) % and (119.92 ± 6.00) %, respectively, while the Au@LA-treated group decreased to (99.16 ± 4.27) % (P < 0.001). After 48 h, the proliferation rate of the Au@LA group further decreased to (70.66 ± 9.24) % (P < 0.001), and after 72 h, it dropped to (63.36 ± 6.93) % (P < 0.001), indicating a sustained anti-proliferative effect of Au@LA.

Apoptosis was detected using Annexin V-FITC/PI double staining flow cytometry (Figures 6B,C). The results showed that the apoptosis rate (Q2+Q3) was (5.22 ± 0.71) % in the NC group (30.51 ± 0.79) % in the LA group (5.47 ± 0.70) % in the Au NPs group; and (53.78 ± 0.50) % in the Au@LA group, which was significantly higher than in the NC, LA, and Au NPs groups (P < 0.001). These results indicate that Au@LA effectively induces HeLa cell apoptosis compared to free LA.

Colony formation assay results (Figures 6D,E) showed that compared to the control group (colony formation rate 55.20% ± 1.59%) and the Au NPs group (55.80% ± 1.25%), the colony formation rate of the free LA group was (38.07 ± 1.14) %, indicating a significant reduction in colony number (P < 0.001). The colony formation rate of the Au@LA treatment group was only (15.20 ± 0.92)%, a more significant decrease compared to the free LA group (P < 0.001). These results demonstrate that Au@LA reduced the proliferation, colony formation, and ability to induce apoptosis of human cervical cancer HeLa cells.

#### 3.4.2 Au@LA induces ferroptosis in HeLa cells

To explore the molecular mechanism of Au@LA-induced HeLa cell death, this study first detected core ferroptosis markers via Western blot. The results showed that compared with the blank control group (NC), Au@LA treatment significantly downregulated the protein levels of the cystine/glutamate antiporter SLC7A11 and glutathione peroxidase 4 (GPX4), while significantly upregulating transferrin receptor 1 (TFR1) (Figures 6F,G). This typical manifestation suggests that Au@LA may exert its effects by triggering ferroptosis. Biochemical index detection further confirmed this hypothesis. Au@LA treatment led to a significant increase in PGE2 secretion (Figure 6H), a significant decrease in intracellular reduced glutathione (GSH) content (Figure 6I), and a significant increase in oxidized glutathione (GSSG) content (Figure 6J), indicating a disruption of intracellular redox homeostasis. Simultaneously, levels of the lipid peroxidation products malondialdehyde (MDA) and 4-hydroxynonenal (4-HNE) were significantly elevated (Figures 6M,N), a hallmark characteristic of ferroptosis. Furthermore, the concentrations of total intracellular iron and ferrous ions (Fe^2+^) were significantly increased (Figures 6K,L), further confirming the activation of ferroptosis. These results indicate that Au@LA triggers ferroptosis in HeLa cells by inhibiting the SLC7A11-GPX4 axis, inducing lipid peroxidation, and promoting iron accumulation. Notably, at the same concentration, Au@LA induced ferroptosis in HeLa cells significantly more effectively than free LA, indicating that nano-modification markedly enhances the ferroptosis-promoting and anti-tumor activity of lauric acid.

**FIGURE 7:**
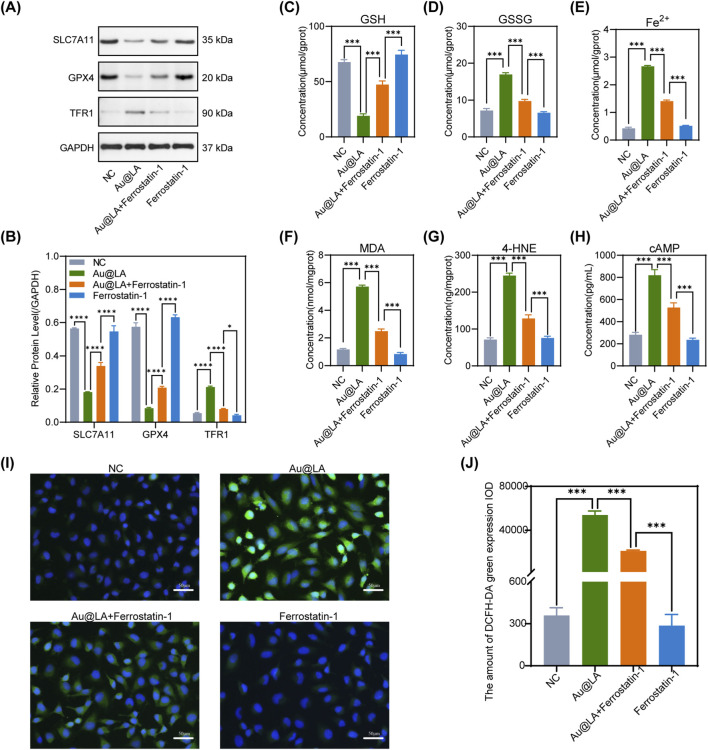
Au@LA induces ferroptosis in HeLa cells, which is reversed by the ferroptosis inhibitor Ferrostatin-1 **(A,B)** Western blot analysis of ferroptosis-related proteins. Representative immunoblots **(A)** and quantitative analysis **(B)** of SLC7A11, GPX4, and TFR1 protein levels, with GAPDH as the internal loading control. The Au@LA-induced downregulation of SLC7A11 and GPX4, and upregulation of TFR1, were significantly reversed by co-treatment with Ferrostatin-1. **(C–H)** Biochemical analysis of ferroptosis markers. Quantitative measurements of intracellular concentrations of GSH **(C)**, GSSG **(D)**, Fe^2+^ **(E)**, MDA **(F)**, 4-HNE **(G)**, and cAMP **(H)** in HeLa cells treated with NC, Au@LA, Au@LA + Ferrostatin-1, or Ferrostatin-1 alone. Ferrostatin-1 significantly rescued the Au@LA-induced alterations in these ferroptosis-related biochemical indicators. **(I,J)** Detection of intracellular reactive oxygen species (ROS) levels using the DCFH-DA probe. Representative fluorescence microscopy images **(I)** and quantitative analysis of DCFH-DA green fluorescence expression **(J)**. The Au@LA-induced elevation of intracellular ROS levels was markedly alleviated by Ferrostatin-1 treatment. Data are presented as mean ± SD (n = 3). Statistical significance is indicated by asterisks (***p < 0.001).

To further confirm that Au@LA-triggered cell death is dependent on ferroptosis, we performed a rescue experiment using the ferroptosis-specific inhibitor Ferrostatin-1 (Fer-1) (HY-100579, MedChemExpress, 2 μM). The rescue experiment showed that when Au@LA was used in combination with Fer-1, the expression of GPX4 and SLC7A11 rebounded, and the downregulation of TFR1 was reversed (Figures 7A,B), indicating that Fer-1 could reverse the regulation of ferroptotic proteins by Au@LA. Concurrently, regarding ferroptosis-related biochemical indices, GSH depletion and GSSG accumulation, ferrous iron (Fe^2+^) overload, and lipid peroxidation were all significantly rescued by Fer-1 (Figures 7C–G), thereby confirming that ferroptosis is the primary mode of cell death triggered by Au@LA. In addition, to investigate early signal transduction during ferroptosis, this study also measured cAMP levels (Figure 7H). The results showed that Au@LA treatment significantly reduced intracellular cAMP content, suggesting its potential involvement in the ferroptosis induction process, an effect effectively reversed by Fer-1 pretreatment. To visually capture the lipid peroxidation process, DCFH-DA fluorescent probe detection was performed, revealing that Au@LA significantly induced intracellular reactive oxygen species (ROS) burst, and this excessive ROS accumulation was effectively inhibited by Fer-1 (Figures 7I,J). These results not only confirm that Au@LA kills tumor cells through the ferroptosis pathway but also suggest that its anti-tumor effect is closely related to the inhibition of PGE2 production.

### 3.5 Au@LA reverses M2-like polarization of THP-1-derived macrophages

**FIGURE 8:**
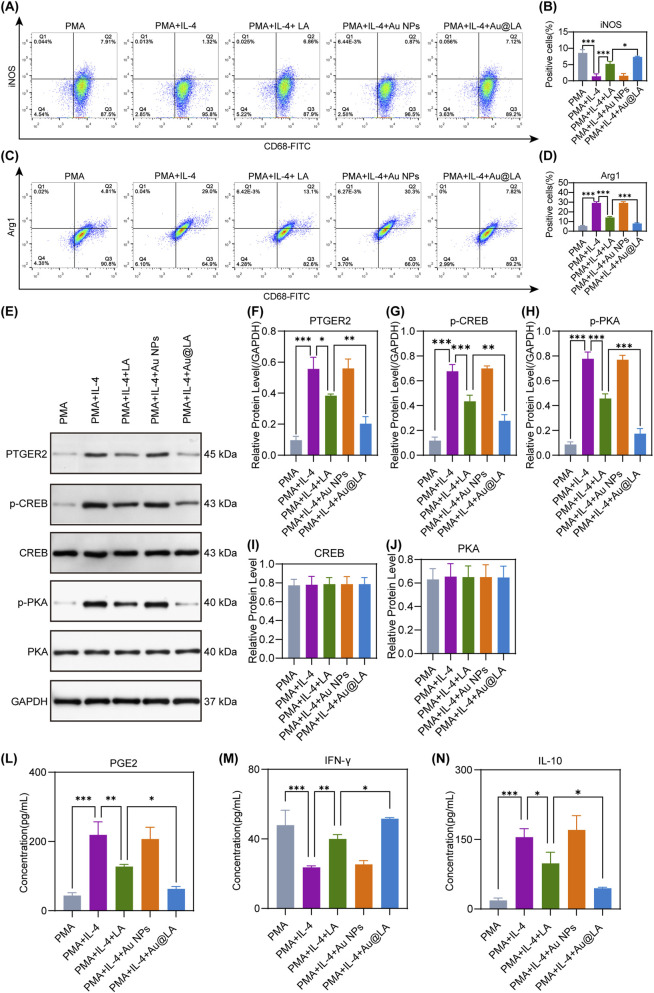
Au@LA reverses IL-4-induced M2-like polarizationof THP-1-derived macrophages via the PGE2/PTGER2/PKA/CREB signaling axis. **(A,B)** Flow cytometry analysis of M1 macrophage marker iNOS expression. Representative dot plots **(A)** and quantitative analysis of iNOS-positive cells **(B)** are shown. THP-1-derived macrophages were treated with PMA, PMA + IL-4, PMA + IL-4 + LA, PMA + IL-4 + Au NPs, or PMA + IL-4 + Au@LA. **(C,D)** Flow cytometry analysis of M2 macrophage marker Arg1 expression. Representative dot plots **(C)** and quantitative analysis of Arg1-positive cells **(D)** are shown. The data indicate that Au@LA significantly reverses IL-4-induced M2-like polarization (decreased iNOS and increased Arg1). **(E)** Western blot analysis of PTGER2, p-CREB, CREB, p-PKA, and PKA protein expression in the indicated groups. GAPDH was used as the internal loading control. **(F–J)** Quantitative analysis of protein expression levels from Western blots. Relative levels of PTGER2 **(F)**, p-CREB **(G)**, p-PKA **(H)**, total CREB **(I)**, and total PKA **(J)** normalized to GAPDH. The phosphorylation levels of CREB and PKA were significantly reduced in the Au@LA treatment group. **(K–N)** ELISA analysis of cytokine concentrations in the cell culture supernatant. Measurements of PGE2 **(K)**, IFN-γ **(L)**, and IL-10 **(M)** levels. Au@LA significantly downregulated the secretion of PGE2 and IL-10 while upregulating the secretion of IFN-γ. Data are presented as mean ± SD (n = 3). Statistical significance is indicated by asterisks (*p < 0.05, **p < 0.01, ***p < 0.001).

Considering the importance of the tumor microenvironment, we assessed the impact of Au@LA on macrophage polarization. After THP-1 cells were differentiated into M0 with PMA (100 nM, 24 h), M2-like polarizationwas induced with IL-4 (20 ng/mL) while simultaneously treating with Au@LA (50 μM) for 48 h. Flow cytometry analysis revealed that IL-4 stimulation drastically increased the proportion of M2-like polarization (CD68^+^/Arg-1^+^), which was significantly reduced by Au@LA treatment (P < 0.001); concurrently, the proportion of M1 macrophages (CD68^+^/iNOS^+^) markedly increased (Figures 8A–D). This indicates that Au@LA effectively reverses the immunosuppressive M2-like polarization to an anti-tumor M1 phenotype.

To deeply elucidate the molecular mechanism of macrophage reprogramming, we focused on the PGE2/EP2/cAMP-PKA signaling axis. Western blot results (Figures 8E–J) showed that compared to the PMA-only group, the PTGER2 protein expression level was significantly elevated in the PMA + IL-4 group (P < 0.001). The addition of Au@LA reduced PTGER2 expression (P < 0.01 vs. PMA + IL-4). Simultaneously, levels of p-CREB and p-PKA were significantly increased in the PMA + IL-4 group, whereas Au@LA treatment significantly inhibited the phosphorylation of CREB and PKA, markedly reducing p-CREB and p-PKA levels (P < 0.01). Free LA exhibited relatively weaker regulatory effects on these proteins. These results indicate that Au@LA can inhibit IL-4-induced EP2 expression and cAMP-PKA signaling pathway activation. ELISA was used to detect the release of inflammatory cytokines in the cell culture supernatant (Figures 8L–N). The results showed that concentrations of PGE2 and IL-10 in the PMA + IL-4 group were significantly higher than those in the PMA-only group (P < 0.001), while IFN-γ was significantly decreased (P < 0.001). After Au@LA treatment, PGE2 concentration decreased from (219.01 ± 37.64) pg/mL to (63.11 ± 6.62) pg/mL (P < 0.001); IFN-γ concentration increased from (23.65 ± 0.89) pg/mL to (51.56 ± 0.69) pg/mL (P < 0.01); and IL-10 concentration decreased from (155.00 ± 18.38) pg/mL to (44.77 ± 2.01) pg/mL (P < 0.001). The downregulating effects of free LA on these cytokines were not as significant as those of Au@LA.

### 3.6 Au@LA inhibits the PGE2/EP2/camp-pka signaling pathway and regulates cytokine release

**FIGURE 9:**
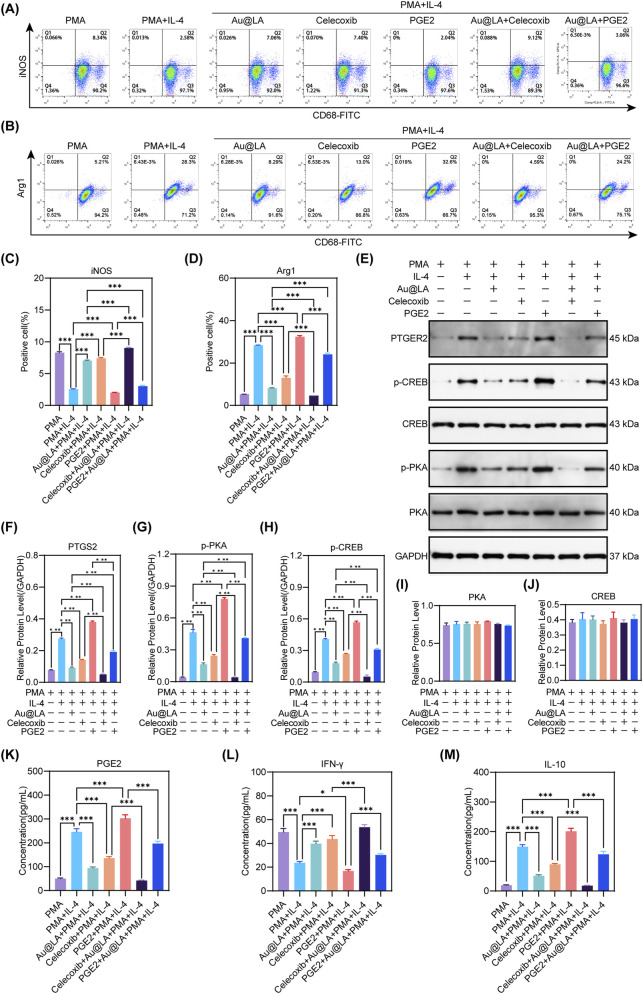
Au@LA reshapes macrophage M1/M2-like polarizationvia the PGE2/EP2/cAMP-PKA signaling axis. **(A,B)** Flow cytometry analysis of M1 marker iNOS **(A)** and M2 marker Arg1 **(B)** expression in CD68^+^ cells across different treatment groups: PMA, PMA + IL-4, Au@LA alone, Celecoxib (a COX-2 inhibitor, used as a positive control), PGE2 alone, and various combination groups. **(C,D)** Quantitative analysis of the percentages of iNOS-positive cells **(C)** and Arg1-positive cells **(D)**. The results indicate that Au@LA significantly reverses IL-4-induced M2 polarization, while the addition of exogenous PGE2 significantly reverses this protective effect, restoring the M2-like polarization. **(E)** Western blot analysis of key signaling pathway proteins (PTGER2, p-CREB, CREB, p-PKA, PKA) across the indicated treatment groups. GAPDH was used as the internal loading control. **(F–J)** Quantitative analysis of relative protein expression levels. Au@LA significantly inhibits the IL-4-induced upregulation of PTGER2 **(F)** and the phosphorylation of PKA **(G)** and CREB **(H)**, whereas exogenous PGE2 reverses these effects. Total levels of PKA **(I)** and CREB **(J)** remain unchanged. **(K–M)** ELISA analysis of PGE2 **(K)**, IFN-γ **(L)**, and IL-10 **(M)** levels in the cell culture supernatant. The results confirm that Au@LA treatment suppresses IL-10 secretion while increasing IFN-γ expression, and this process is regulated by the PGE2 signaling axis, as these effects are rescued by the addition of exogenous PGE2. Data are presented as mean ± SD (n = 3). Statistical significance is indicated by asterisks (*p < 0.05, **p < 0.01, ***p < 0.001).

To further validate the central role of the PGE2/EP2/cAMP-PKA signaling axis in Au@LA-mediated macrophage reprogramming, rescue experiments were conducted using pharmacological blockade (COX-2 inhibitor Celecoxib) and exogenous supplementation (PGE2). Flow cytometry results (Figures 9A,B) showed that the proportion of M2-like polarization (CD68^+^/iNOS^−^, i.e., CD68-positive and iNOS-negative population) induced by PMA + IL-4 significantly increased to 13.3%, while Au@LA treatment markedly suppressed this process, reducing the M2 proportion to 3.5%. When cells were pretreated with Celecoxib (a COX-2 inhibitor, blocking PGE2 synthesis, HY-100579,MedChemExpress, 2 μM) for 1 h, even with the addition of Au@LA, the M2 macrophage proportion remained at a very low 2.8%; conversely, when exogenous PGE2 (HY-101952,MedChemExpress, 10 μM)was supplemented alongside Celecoxib for 48 h, the M2 macrophage proportion significantly rebounded to 7.9%, approaching the model group level. Flow cytometric results for the M2 marker Arg-1 (Figures 9A–D) exhibited a completely consistent trend: Au@LA significantly inhibited IL-4-induced Arg-1 expression, and PGE2 supplementation significantly reversed this inhibitory effect. Western blot results (Figures 9E–J) further validated the above phenotypic changes at the protein phosphorylation level. Compared to the PMA + IL-4 alone group, Au@LA treatment significantly reduced the protein expression levels of the EP2 receptor (PTGER2) and the downstream signaling molecules p-CREB and p-PKA. The Celecoxib treatment group demonstrated an inhibitory effect similar to Au@LA, whereas the exogenous PGE2 supplementation group significantly reversed the protein downregulation induced by Au@LA, restoring the expression of PTGER2, p-CREB, and p-PKA. The levels of total CREB and PKA proteins showed no significant changes among the groups, indicating that Au@LA mainly affects receptor expression and downstream kinase phosphorylation activation. ELISA results (Figures 9K–M) quantitatively analyzed key inflammatory factors in the cell supernatant. In terms of PGE2 concentration, Au@LA treatment significantly decreased it, while the PGE2 rescue experiment markedly increased its concentration. Regarding functional cytokines, Au@LA significantly increased the secretion of the pro-inflammatory/anti-tumor factor IFN-γ and decreased the secretion of the anti-inflammatory/pro-tumor factor IL-10. Exogenous PGE2 supplementation significantly attenuated the enhancing effect of Au@LA on IFN-γ and its inhibitory effect on IL-10. The modulation amplitude of these cytokines by free LA was much smaller than that of Au@LA nanoparticles.

In summary, Au@LA reduces endogenous PGE2 synthesis by inhibiting COX-2/PTGS2, thereby blocking the activation of the EP2/cAMP-PKA/CREB signaling pathway, significantly decreasing the release of immunosuppressive cytokines PGE2 and IL-10 while increasing the level of the pro-inflammatory cytokine IFN-γ. This effectively reverses the IL-4-induced immunosuppressive microenvironment, ultimately driving macrophage polarization from the immunosuppressive M2-like polarization to the immunostimulatory M1 phenotype.

## 4 Discussion

Cervical cancer is one of the common malignancies in women. Although surgery, chemoradiotherapy, and immunotherapy have significantly improved the prognosis of some patients, tumor recurrence, drug resistance, and the immunosuppressive tumor microenvironment remain major factors limiting treatment efficacy (Tewari, 2025). Therefore, developing novel therapeutic strategies that possess both tumor cell-killing ability and immune microenvironment modulation is of great significance (Huang et al., 2025). Based on network pharmacology screening of key active components, core targets, and potential pathways of Crocus sativus L. against cervical cancer, this study further constructed lauric acid-modified gold nanoparticles (Au@LA) and systematically evaluated their effects on cervical cancer HeLa cell proliferation, apoptosis, ferroptosis, and macrophage polarization. The results indicated that lauric acid may be one of the important active components of Crocus sativus L. in exerting anti-cervical cancer effects, with PTGS2 identified as a key candidate target in silico.Au@LA significantly enhanced the anti-tumor activity of lauric acid, inhibited HeLa cell proliferation and colony formation, induced apoptosis and ferroptosis, and reversed M2-like polarization of THP-1-derived macrophages by regulating the PGE2/EP2/cAMP-PKA signaling axis, suggesting that Au@LA has potential anti-cervical cancer and immunomodulatory value.

Initially, network pharmacology analysis revealed extensive overlap between the potential targets of Crocus sativus L. active components and cervical cancer disease targets, suggesting that Crocus sativus L. may exert anti-tumor effects through multi-component, multi-target, multi-pathway synergy. GO functional enrichment results showed that the intersection targets were not only involved in tumor-related processes such as inflammatory response, post-translational protein modification, and G protein-coupled receptor signal transduction but were also significantly enriched in long-chain fatty acid metabolism, fatty acid oxidation, and fatty acid metabolic processes. This suggests that lipid metabolism reprogramming may be an important mechanism by which Crocus sativus L. intervenes in cervical cancer. Tumor cells typically exhibit abnormally active fatty acid synthesis, uptake, and oxidation to meet the demands for membrane lipid synthesis, energy supply, and signaling molecule generation required for rapid proliferation (Quan et al., 2022; Mehta et al., 2023). Abnormal lipid metabolism is also closely related to tumor invasion, drug resistance, and immune escape (Zhang et al., 2021). Therefore, targeting fatty acid metabolism-related pathways may provide a new entry point for cervical cancer therapy.

KEGG enrichment analysis showed that the intersection targets were mainly enriched in pathways such as pathways in cancer, neuroactive ligand-receptor interaction, xenobiotic metabolism, lipid and atherosclerosis, chemical carcinogenesis, calcium signaling pathway, and AGE-RAGE signaling pathway. Pathways in cancer and chemical carcinogenesis directly reflect the potential intervention of Crocus sativus L. components in tumor development and progression. The calcium signaling pathway is closely related to cell proliferation, apoptosis, mitochondrial function, and oxidative stress (Rabchevsky et al., 2020; Ren et al., 2021); the AGE-RAGE signaling pathway is associated with chronic inflammation, oxidative stress, and tumor microenvironment remodeling (Sionov, 2021; Dobrucki et al., 2024). Furthermore, the lipid and atherosclerosis pathway involves numerous molecules related to inflammation, lipid oxidation, and macrophage function regulation, showing good consistency with the observed changes in lipid peroxidation and macrophage polarization in subsequent experiments.

PPI network analysis further identified core targets including PTGS2, STAT3, MAPK1, MAPK3, AKT1, JUN, TNF, IL6, EGFR, and VEGFA. These targets are all closely related to tumor cell proliferation, inflammatory response, angiogenesis, immune regulation, and therapy resistance (Meng and Xia, 2011; Sgrignani et al., 2018; Hua et al., 2021; Gong T. et al., 2022; Liu and Miao, 2024; Lee et al., 2025; Nishida and Andoh, 2025; Shen et al., 2026). Among them, PTGS2 encodes cyclooxygenase-2 (COX-2), a key rate-limiting enzyme in the conversion of arachidonic acid to prostaglandins, particularly PGE2 (Anamthathmakula and Winuthayanon, 2021). The COX-2/PGE2 axis is aberrantly activated in various tumors, promoting tumor cell proliferation, inhibiting apoptosis, enhancing invasion and metastasis, and inducing the formation of an immunosuppressive microenvironment (Gong Z. et al., 2022; Balamurugan et al., 2023; Qi et al., 2023). Studies have shown that high COX-2 expression is associated with cervical cancer progression, chemoradiotherapy resistance, and poor prognosis (Xiao et al., 2021; García-Quiroz et al., 2022). Therefore, PTGS2 being the node with the highest degree value in the PPI network in this study suggests it may be a key candidate target for Crocus sativus L. intervention in cervical cancer.

In the “component-target-pathway-disease” network, active components such as lauric acid, kaempferol, and isorhamnetin were located at relatively core positions. Flavonoids like kaempferol and isorhamnetin have been extensively studied for their antioxidant, anti-inflammatory, pro-apoptotic, and anti-tumor effects (Martiniakova et al., 2024; Sergiel, 2024a; 2024b), while the anti-tumor potential of lauric acid, a medium-chain saturated fatty acid, has also gained attention in recent years (Pan et al., 2024; Jiménez-Franco et al., 2026). Lauric acid can affect cell membrane structure, lipid metabolism, mitochondrial function, and inflammatory signaling, and may induce cell death by altering lipid homeostasis in tumor cells (Mallick and Mondal, 2023; Figueiredo et al., 2025). The molecular docking results showed that lauric acid can stably bind to PTGS2 *in silico* with a binding energy of −4.774 kcal/mol, forming hydrophobic and van der Waals interactions with residues including THR192, ASN368, TRP373, ALA188, TYR371, GLN189, and PHE196. Moreover, SPR analysis confirmed this direct interaction with a high binding affinity (KD = 6.05 × 10^−8^ M), providing robust experimental evidence for the lauric acid-PTGS2 interaction. Combined with the network analysis results, this supports the possibility that lauric acid participates in anti-cervical cancer effects by modulating PTGS2-related pathways.

Natural products have garnered significant attention due to their broad bioactivities. However, free lauric acid suffers from poor water solubility, limited bioavailability, and low cellular uptake efficiency, which may restrict its biological effects (Panbachi et al., 2024). Therefore, this study employed gold nanoparticles as carriers to prepare lauric acid-functionalized nanoparticles (Au@LA) to improve the delivery efficiency and cellular potency of lauric acid. Gold nanoparticles offer advantages such as controllable particle size, easy surface modification, good biocompatibility, and stable optical properties, making them widely used in drug delivery, tumor diagnosis, and therapy research (Poulie et al., 2022). In this study, Au NPs were prepared by the sodium citrate reduction method, followed by amination with cysteamine and subsequent coupling with lauric acid. UV-visible absorption spectroscopy showed a slight red shift of the SPR peak from 529 nm for Au NPs to 531 nm for Au@LA. Zeta potential changed from −30.3 mV for Au NPs to +14.8 mV for Au-NH_2_, and then decreased to +8.89 mV for Au@LA, indicating successful stepwise modification with cysteamine and lauric acid. TEM and DLS results showed an increase in particle size for Au@LA with some degree of aggregation, but the PDI remained relatively low, suggesting acceptable overall dispersion. These physicochemical characterization results confirm the successful construction of Au@LA, providing the material basis for subsequent biological experiments.

In cell function assays, Au@LA exhibited significant concentration-dependent inhibition of HeLa cells, with a 48 h IC_50_ of approximately 52.29 µM, whereas free LA and Au NPs at the same concentration ranges had weak effects on HeLa cells. More importantly, Au@LA showed no significant inhibitory effect on normal cervical immortalized epithelial Ect1/E6E7 cells or THP-1 cells, suggesting a degree of tumor selectivity and good biocompatibility, which is crucial for reducing systemic toxicity. Time-proliferation curves further demonstrated that Au@LA significantly inhibited HeLa cell proliferation at 48 h and 72 h, indicating sustained anti-proliferative effects. Colony formation assays also confirmed that Au@LA significantly reduced the long-term proliferation and clonogenic capacity of HeLa cells, with superior effects compared to free LA. This may be attributed to the nano-carrier promoting the uptake of lauric acid by tumor cells, enhancing intracellular retention, or altering its subcellular distribution.

Apoptosis is one of the important mechanisms by which anti-tumor drugs induce cancer cell death (Ichim and Tait, 2016). The Annexin V-FITC/PI double staining results in this study showed that the total apoptotic rate of HeLa cells was significantly increased after Au@LA treatment, markedly higher than that in the free LA and Au NPs groups, indicating that nano-modification significantly enhances the apoptosis-inducing ability of lauric acid. Although this study did not further detect classic apoptotic indicators such as Caspase family, Bax/Bcl-2 ratio, or mitochondrial membrane potential, the flow cytometry results preliminarily demonstrate that Au@LA can effectively induce programmed cell death in HeLa cells. Given that abnormal fatty acid metabolism, oxidative stress, and mitochondrial dysfunction can all induce apoptosis, Au@LA may trigger apoptotic programs by interfering with lipid homeostasis and redox balance in tumor cells.

Beyond apoptosis, this study focused on the regulatory effect of Au@LA on ferroptosis. Ferroptosis is a form of regulated cell death characterized by iron dependence and the accumulation of lipid peroxidation, typically accompanied by increased intracellular free iron, glutathione depletion, GPX4 inactivation, and elevated levels of lipid peroxidation products such as MDA and 4-HNE(Lei et al., 2024). In recent years, inducing ferroptosis has been recognized as an important strategy to overcome tumor drug resistance and eliminate apoptosis-resistant tumor cells (Cañeque et al., 2025). Aberrant activation of the SLC7A11-GSH-GPX4 axis in cervical cancer cells enhances antioxidant capacity, protects against lipid peroxidation, and promotes tumor survival (Shang et al., 2026). Therefore, inhibiting SLC7A11 and GPX4 may effectively reduce tumor cell resistance to ferroptosis.

This study found that Au@LA significantly downregulated the protein expression of SLC7A11 and GPX4 in HeLa cells, while upregulating TFR1 expression. SLC7A11 is a key component of system Xc^−^, responsible for cysteine uptake and maintaining GSH synthesis (Tu et al., 2021); GPX4 utilizes GSH to reduce lipid peroxides and is the core enzyme inhibiting ferroptosis (Chen et al., 2023). Upregulation of TFR1 promotes iron uptake, increasing the intracellular iron pool and providing conditions for the Fenton reaction and lipid peroxidation (Floros et al., 2022). Therefore, the inhibition of the SLC7A11-GPX4 axis and upregulation of TFR1 induced by Au@LA together drive the occurrence of ferroptosis. Consistently, Au@LA significantly reduced GSH levels, increased GSSG levels, and elevated Fe^2+^, total iron, MDA, and 4-HNE contents, indicating disruption of intracellular redox homeostasis and markedly enhanced iron-dependent lipid peroxidation. Crucially, the ferroptosis inhibitor Ferrostatin-1 reversed these changes, confirming that ferroptosis is the primary mode of cell death. These results collectively support that Au@LA exerts anti-HeLa cell effects by inducing ferroptosis.

Network pharmacology analysis positioned PTGS2 (cyclooxygenase-2) as a core target in the action network of Crocus sativus L. active components, and molecular docking combined with SPR experiments confirmed that lauric acid stably binds to the active pocket of PTGS2 with high affinity. Notably, Au@LA treatment exerted opposite effects on PGE2 levels in the two cellular models: it significantly increased PGE2 in the supernatant of HeLa cells, whereas it markedly suppressed PGE2 release in IL-4-induced M2-like polarization. This bidirectional regulation of the same lipid mediator initially appears paradoxical. However, when interpreted in the context of cell type-specific biology and the distinct experimental settings, this discrepancy becomes mechanistically plausible. N HeLa cells, the elevation of PGE2 does not indicate a classical “activation” of the PTGS2/PGE2 axis to promote tumor growth. Instead, it is most likely a consequence of Au@LA-induced ferroptosis. Ferroptosis is characterized by iron-dependent lipid peroxidation, leading to the accumulation of reactive aldehydes such as 4-hydroxynonenal (4-HNE) and malondialdehyde (MDA). Multiple studies have demonstrated that ferroptotic stress can upregulate PTGS2 (COX-2) expression and subsequently increase PGE2 production as part of a cellular stress response, rather than a pro-survival signal (Zhou et al., 2021; Liu et al., 2023). In our study, Au@LA treatment significantly increased MDA, 4-HNE, and Fe^2+^ levels while downregulating SLC7A11 and GPX4 in HeLa cells, confirming active ferroptosis. Under these conditions, elevated PGE2 likely reflects oxidative injury and lipid peroxidation-induced COX-2 activation, not a direct agonistic effect of lauric acid on PTGS2. Critically, the ferroptosis inhibitor Ferrostatin-1 (Fer-1) not only reversed the protein changes in SLC7A11, GPX4, and TFR1, and restored GSH levels, but also fully rescued the Au@LA-induced decline in intracellular cAMP—a key second messenger downstream of PGE2. The fact that cAMP levels dropped despite elevated PGE2 suggests that ferroptosis disrupts the PGE2/EP2/cAMP signaling axis, possibly through oxidative damage to receptors or adenylate cyclase, and Fer-1 restores this axis by blocking ferroptosis. Thus, the increase in PGE2 is a ferroptosis-associated stress phenomenon, functionally disconnected from pro-survival cAMP signaling. In contrast, in IL-4-induced M2-like polarization, Au@LA does not trigger ferroptosis (no significant lipid peroxidation or GSH depletion was observed). Instead, it directly interferes with the signaling cascade that drives M2 polarization. Our data show that Au@LA markedly reduces the expression of the PGE2 receptor EP2 (PTGER2), decreases intracellular cAMP levels, and suppresses the phosphorylation of its downstream effectors PKA and CREB. The PGE2/EP2/cAMP-PKA axis is known to promote M2-like polarizationand the secretion of immunosuppressive cytokines such as IL-10 (Stadtmauer and Wagner, 2022; Wang et al., 2022). By downregulating EP2 and inhibiting COX-2 activity, Au@LA disrupts this autocrine/paracrine loop, leading to decreased PGE2 production and the subsequent reversal of M2 polarization. This is fundamentally different from the ferroptosis-associated PGE2 elevation in cancer cells. Pharmacological rescue experiments further validated this mechanistic distinction: the COX-2 inhibitor Celecoxib mimicked the effects of Au@LA, significantly reducing M2 polarization, whereas exogenous PGE2 supplementation reversed the Au@LA-induced suppression of PTGER2, p-PKA, and p-CREB, restored Arg-1 expression, and increased the M2 macrophage proportion. These bidirectional rescue results conclusively demonstrate that Au@LA acts through the PGE2/EP2/cAMP-PKA signaling axis to reprogram macrophages. Thus, the opposite direction of PGE2 changes arises from cell type-specific pathophysiological contexts: in HeLa cells, Au@LA induces lethal ferroptosis with secondary PGE2 elevation as a stress marker, confirmed by the complete rescue of downstream cAMP signaling by Fer-1; in macrophages, Au@LA directly inhibits the EP2/cAMP/PKA signaling axis, which secondarily reduces PGE2 levels and reverses M2 polarization, verified by both pharmacological inhibition and exogenous PGE2 add-back experiments. This dual mode of action is not contradictory but reflects the ability of lauric acid (and its nanoformulation) to engage different molecular hubs depending on the cellular environment—promoting ferroptotic death signaling in tumor cells while blocking immunosuppressive signaling in immune cells. Future studies using cell-specific PTGS2 or EP2 knockout models could further dissect these distinct mechanisms, but the current results already provide a coherent and literature-supported explanation for the observed discrepancy.

Tumor-associated macrophages are an important component of the cervical cancer microenvironment (Guo F. et al., 2021; Wang et al., 2024). M1 macrophages typically secrete pro-inflammatory factors and exert anti-tumor immune effects (Kang and Kumanogoh, 2020; Chabeli et al., 2022; Arnosa-Prieto et al., 2024), while M2-like polarization are associated with immunosuppression, angiogenesis, tumor invasion, and metastasis (Ohashi et al., 2020; Fan et al., 2024; Sezginer and Unver, 2024). Increased infiltration of M2-like polarization in cervical cancer tissues is often correlated with tumor progression and poor prognosis (Kim et al., 2020; Tan et al., 2022; Wang et al., 2022). Therefore, reprogramming M2-like polarization toward an M1 phenotype is an important strategy to improve the tumor immune microenvironment. In this study, PMA was used to induce THP-1 differentiation into macrophages, and IL-4 was used to induce M2 polarization. Results showed that Au@LA significantly reduced the proportion of CD68^+^/Arg-1^+^ M2 cells and increased the proportion of CD68^+^/iNOS^+^ M1 cells, indicating that Au@LA can effectively reverse IL-4-induced M2-like polarization and promote the transition of macrophages to an anti-tumor M1 phenotype. Compared to free LA, the effect of Au@LA was more pronounced, further demonstrating that nano-delivery enhances the immunomodulatory activity of lauric acid.

Mechanistically, this study found that Au@LA inhibited the expression of the EP2 receptor PTGER2 in macrophages, decreased intracellular cAMP levels, and reduced the phosphorylation levels of PKA and CREB. PGE2 activates the cAMP-PKA-CREB signaling pathway through the EP2 receptor, which can promote the release of immunosuppressive factors such as IL-10, inhibit the production of pro-inflammatory cytokines, and drive macrophage polarization toward the M2-like polarization (Stadtmauer and Wagner, 2022; Wang et al., 2022). The reduction of PTGER2 expression, cAMP levels, and PKA/CREB phosphorylation by Au@LA suggests that it may remodel macrophage function by blocking the PGE2/EP2/cAMP-PKA signaling pathway. ELISA results further supported this mechanism: Au@LA significantly reduced the release of PGE2 and IL-10 while increasing IFN-γ levels. Pharmacological blockade with the COX-2 inhibitor Celecoxib mimicked the effects of Au@LA, whereas exogenous PGE2 supplementation reversed them, conclusively validating the central role of this signaling axis in Au@LA-mediated macrophage reprogramming. IL-10 is a classic immunosuppressive factor that inhibits antigen presentation and T cell effector function (Zheng et al., 2020; Guo et al., 2025); IFN-γ helps promote macrophage M1 activation and enhance anti-tumor immunity (Guo Q. et al., 2021; Zhang et al., 2022). Therefore, Au@LA may simultaneously play a dual role as a “cell killer” and “immune modulator”. The damage-associated molecular patterns (DAMPs) released from tumor cells undergoing Au@LA-induced ferroptosis may further activate the immune system, potentially synergizing with macrophage phenotype remodeling to achieve more effective inhibition of cervical cancer.

Integrating the results of this study, the following mechanistic model can be proposed: Lauric acid, an active component of Crocus sativus L., can target or modulate PTGS2-related lipid metabolism and PGE2 signaling. Loading lauric acid onto the surface of gold nanoparticles (Au@LA) improves its cellular delivery efficiency and bioactivity. In HeLa cells, Au@LA inhibits the SLC7A11-GPX4 antioxidant defense axis, upregulates TFR1, promotes iron accumulation, leads to accumulation of lipid peroxidation products such as MDA and 4-HNE, and ultimately induces ferroptosis, while also promoting apoptosis and inhibiting colony formation. The concurrent suppression of PGE2 and cAMP further facilitates this ferroptotic process. In macrophages, Au@LA inhibits PGE2/EP2/cAMP-PKA-CREB signaling, reduces the secretion of immunosuppressive factors such as IL-10, and increases IFN-γ levels, thereby reversing M2-like polarizationand promoting the formation of an anti-tumor M1 phenotype. This dual mechanism gives Au@LA the potential for both “direct tumor cell killing” and “remodeling of the immune microenvironment.”

This study has several innovations. First, network pharmacology was used to identify lauric acid and the PTGS2 axis as a potentially key “component-target” pair from the multi-component system of Crocus sativus L., providing a basis for modern research on active ingredients of traditional Chinese medicine. Second, to address the poor water solubility and bioavailability of lauric acid, an Au@LA nano-delivery system was constructed and confirmed by various physicochemical characterizations. Third, the anti-cervical cancer effects of Au@LA were systematically evaluated from the perspectives of tumor cell ferroptosis and macrophage polarization, suggesting not only direct cytotoxicity but also potential for immune microenvironment modulation.

Compared to conventional gold nanoparticle-based drug delivery systems that rely on loaded chemotherapeutics, Au@LA exploits the intrinsic bioactivity of lauric acid, potentially reducing the risk of drug resistance and off-target toxicity associated with traditional cytotoxic agents. Unlike many synthetic ferroptosis inducers (e.g., erastin, RSL3) that target system Xc^−^ or GPX4 directly but suffer from poor pharmacokinetics, Au@LA offers a natural product-based alternative. Moreover, while several nanoformulations have been reported to induce ferroptosis or repolarize macrophages, few achieve both effects simultaneously. The dual functionality of Au@LA—triggering ferroptosis in tumor cells while reprogramming M2-like macrophages toward an M1-like phenotype—distinguishes it from most existing nanomedicines. However, direct head-to-head comparisons with benchmark ferroptosis inducers (e.g., erastin, sulfasalazine) or macrophage-modulating agents (e.g., clodronate liposomes) are warranted in future studies to quantitatively establish its relative efficacy.

However, this study has several limitations. First, the network pharmacology results rely on public databases and prediction algorithms; the large number of disease targets may include genes with varying degrees of association with cervical cancer. Subsequent studies should use stricter screening strategies or clinical sample validation for key targets. Second, although molecular docking and SPR experiments have confirmed the direct binding between lauric acid and PTGS2, these *in vitro* findings do not fully prove direct targeting of PTGS2 within a cellular context. Further validation using cellular thermal shift assays (CETSA) or target knockdown/overexpression experiments is still needed. Third, while the assessment of ferroptosis included indicators such as SLC7A11, GPX4, TFR1, iron ions, GSH/GSSG, MDA, and 4-HNE, and the induction of ferroptosis was confirmed by rescue experiments using the ferroptosis inhibitor Ferrostatin-1, additional validation with other ferroptosis inhibitors (e.g., Liproxstatin-1) or the iron chelator DFO would further strengthen this conclusion. Fourth, this study mainly used HeLa cells and THP-1-derived macrophages; further validation in additional cervical cancer cell lines such as SiHa, CaSki, C33A, as well as in primary macrophages or tumor organoid models, is necessary. Fifth, the *in vivo* distribution, pharmacokinetics, long-term safety, immunogenicity, and anti-tumor efficacy of Au@LA have not been evaluated. Future systematic studies in cervical cancer animal models are needed.

Furthermore, the covalent nature of the lauric acid-gold linkage has not been directly confirmed by spectroscopic methods (e.g., FTIR, XPS); thus, the formulation is described as lauric acid-modified Au NPs, and the preparation process of Au@LA has room for further optimization. In this study, the zeta potential of Au@LA was lower than that of Au NPs, TEM showed some degree of aggregation, and the DLS size was significantly larger. Although the PDI suggested acceptable dispersion, *in vivo* applications, particle size, surface charge, and stability significantly affect blood circulation time, tumor accumulation efficiency, cellular uptake, and clearance by the reticuloendothelial system. Future improvements could include the introduction of PEGylation, tumor-targeting ligands, or optimization of lauric acid grafting density to enhance the colloidal stability and targeted delivery capabilities of the nanoparticles. Additionally, the release kinetics of lauric acid from Au@LA, stability under different pH conditions, and the potential contribution of the gold nanoparticle carrier itself to ferroptosis and immune modulation should be further clarified. This study compared Au@LA with free lauric acid and unmodified Au NPs, demonstrating the superior activity of the lauric acid-modified formulation. However, direct comparisons with established ferroptosis inducers (e.g., erastin, RSL3, or sulfasalazine) or known macrophage repolarizing agents (e.g., clodronate liposomes, CpG oligodeoxynucleotides) were not performed. Such comparisons would be valuable to benchmark Au@LA against existing strategies in terms of potency, selectivity, and dual functionality. Future studies should include these reference compounds to quantitatively position Au@LA within the current therapeutic landscape.

In conclusion, based on network pharmacology, molecular docking, SPR validation, nanomaterial construction, and comprehensive *in vitro* functional validation including inhibitor rescue and signaling pathway reconstitution experiments, this study reveals the potential role of the Crocus sativus L. active component lauric acid in the treatment of cervical cancer and demonstrates that Au@LA can significantly enhance the anti-tumor bioactivity of lauric acid. Au@LA exerts a dual anti-tumor effect by inducing apoptosis and ferroptosis in HeLa cells, while simultaneously inhibiting PGE2/EP2/cAMP-PKA signaling and reversing M2 macrophage polarization. These findings provide experimental evidence for the nano-delivery of lauric acid and the anti-cervical cancer effects of Crocus sativus L. active components, and offer a new strategy for developing novel nano-therapeutic approaches that combine ferroptosis induction and immune microenvironment remodeling.

## Data Availability

The original contributions presented in the study are included in the article/Supplementary Material, further inquiries can be directed to the corresponding authors.
